# Quality of life and treatment-related burden during ocular proton therapy: a prospective trial of 131 patients with uveal melanoma

**DOI:** 10.1186/s13014-021-01902-6

**Published:** 2021-09-08

**Authors:** Johannes Gollrad, Christopher Rabsahl, Aline-Isabel Riechardt, Jens Heufelder, Andrea Stroux, Ute Goerling, Antonia Joussen, Volker Budach, Dirk Boehmer

**Affiliations:** 1 Department of Radiation Oncology, Charité – Universitätsmedizin Berlin, Berlin, Germany; 2 Department of Ophthalmology, Charité – Universitätsmedizin Berlin, Berlin, Germany; 3 Institute of Biometry and Clinical Epidemiology, Charité – Universitätsmedizin Berlin, Berlin, Germany; 4 Department of Psycho-Oncology, CCCC, Charité – Universitätsmedizin Berlin, Berlin, Germany

**Keywords:** Uveal melanoma, Proton therapy, Quality of life

## Abstract

**Background:**

Proton beam therapy is a well-established treatment option for patients with uveal melanoma (UM). The treatment procedure, in general, includes placing radiopaque clips to ensure exact eye-positioning during radiotherapy, followed by the delivery of proton irradiation.

The short-term burden associated with proton therapy in patients with UM has rarely been addressed. In this prospective study, we investigated the physiological and psychological aspects of proton therapy that might affect the well-being of patients during the different stages of treatment.

**Methods:**

During the treatment procedure, we conducted longitudinal assessments of the Quality of life (QOL), organ-specific symptoms, and psychological aspects in patients with UM with three questionnaires (EORTC QLQ-C30, EORTC QLQ-OPT30, and GAD-7). Patients completed questionnaires before clip surgery (T0), before proton therapy (T1), after completing treatment (T2), and three months after treatment completion (T3). We also collected data on tumor characteristics and socio-demographics to identify potential risk factors associated with high treatment burdens.

**Results:**

We prospectively included 131 consecutive patients. Questionnaire data showed a significant, temporary decline in global QOL and an increase in eye-related symptoms, as a result of the clip surgery (T0–T1). After treatment completion (T2), global QOL improved gradually, and none of the eye-related symptoms significantly deteriorated over the course of proton therapy. The global QOL returned to baseline levels three months after treatment (T3). We identified baseline anxiety as an independent risk factor for experiencing an acute treatment-related burden. Furthermore, we found interactions between GAD7 and patient sex showing that anxiety had a more pronounced effect on QOL outcome in female patients.

**Conclusion:**

The short-term treatment-related burden of ocular proton therapy appeared to be largely associated with the preceding clip surgery, rather than the irradiation procedure. We found that anxiety was strongly associated with experiencing QOL issues during the treatment procedure. Our findings could contribute to the development of future strategies for improving the treatment process and psycho-oncologic patient care.

**Supplementary Information:**

The online version contains supplementary material available at 10.1186/s13014-021-01902-6.

## Introduction

Uveal melanoma (UM) is a rare disease, with a maximum incidence of 8 per million in Europe, but it represents the most common primary cancer of the eye [1]. For decades, proton beam therapy has been widely used as the primary treatment for non-metastasized UM. This treatment is highly effective in terms of local control and long-term globe preservation [2, 3]. Alternative effective treatment approaches include brachytherapy and enucleation [4, 5]. Although, currently, the use of enucleation is often restricted to very advanced tumors, brachytherapy is of limited use for peripapillary tumors and highly prominent tumors. Despite the lack of comparative prospective evidence, these three treatment strategies have shown acceptable results in terms of local control and overall survival. Thus, treatment-related quality of life (QOL) has become a crucial issue in these patients.

Worldwide, only a few dedicated proton facilities provide the particular beam specifications necessary for adequate treatment of UM [6]. In general, ocular proton therapy comprises two major steps. First, radiopaque clips are placed surgically onto the sclera, which allows an x-ray verification of the patients’ eye position immediately before each proton treatment. After clip surgery, proton therapy is delivered in four sessions on consecutive days.

The QOL of patients after UM treatment has been investigated in numerous studies over the past few years, and different treatment methods have been compared [7–15]. However, studies on the pre-treatment QOL, with a focus on the treatment procedure, are rare. Moreover, proton therapy-specific side effects are frequently not addressed.

Hope-Stone et al. reported that the QOL in 411 patients treated for UM was similar to normative data during a two-year follow-up time. However, in that study, the first assessment started 6 months after treatment, and pre-treatment data were not available [13]. Other recent studies have addressed the pre-treatment and peri-interventional QOL status of patients with UM. Barker et al. studied the QOL in 201 patients with UM before undergoing brachytherapy with the EORTC QLQ-C30 questionnaire and its ophthalmic oncology QOL module (QLQ-OPT30) [9]. The majority of patients reported ophthalmic symptoms, like “ocular irritation” (66%) and “vision impairment” (81%), and 41% reported “worry about disease recurrence”. The same set of questionnaires was used by Suchocka-Capuano et al. to evaluate QOL in 69 patients, prior to and one month after radiotherapy; and 63 of those patients received proton therapy [7]. They observed a significant decline in the subscale “social functioning”, but anxiety symptoms improved one month after treatment, and global QOL remained stable. Van Beek et al. compared QOL outcomes in 113 patients treated with either stereotactic radiotherapy or enucleation [10]. Two months after treatment, the stereotactic radiotherapy group demonstrated improved “role functioning” and “emotional functioning”, compared to the pre-treatment assessment.

The present study aimed to refine our understanding of the ophthalmic symptoms and the psychological impact associated with ocular proton therapy procedures, including the pre-therapeutic clip surgery.

## Methods and materials

Between May 2019 and January 2020, 183 patients with non-metastatic UM were admitted to the department of ophthalmology at XXX and considered eligible for proton treatment. Eligibility criteria for proton therapy were central tumor location or a tumor prominence exceeding 5 mm. Exclusion criteria were: tumor confined to the iris, tumor recurrence and insufficient German language skills. These patients were screened for participation in the present prospective QOL study. The study was approved by the XXX Ethics Committee, and it was conducted according to the Declaration of Helsinki and the International Council on Harmonization of Technical Requirements for Registration of Pharmaceuticals for Human Use—Good Clinical Practice.

Before starting radiotherapy, all patients underwent a surgical intervention. During surgery, 4 tantalum clips were sutured onto the sclera. Subsequently, proton beam therapy was delivered on 4 consecutive days; each day, a single fraction was delivered at a dose of 15 Cobalt Gray Equivalent (CGE). During each radiotherapy session, all patients were immobilized with a thermoplastic mask equipped with a bite block. The patient’s eye was directed into the desired orientation by a mobile fixation light. Custom-made lid retractors withdrew the patient’s eye lids from the beam field. Correct patient positioning was ensured with an X-ray verification of the precomputed clip-arrangement. More detailed information on the technical procedure was published previously [16, 17].

After providing written informed consent, all patients completed a set of questionnaires at four particular time points: before clip-surgery (T0), between clip-surgery and proton therapy (T1), after proton therapy (T2), and three months after treatment (T3), as indicated in Table 1.

**Table 1:** Summary of all questionnaires completed by patients, at different times relative to treatment (T0-T3)

Times that questionnaires were handed out	T		Questionnaires	
Pre-treatment status before clip surgery	T0		QLQ-C30, QLQ-OPT30	
After clip-surgery, before proton beam radiotherapy	T1		QLQ-C30, QLQ-OPT30, GAD7	
After completion of proton radiotherapy	T2		QLQ-C30, QLQ-OPT30	
Three months after completion of proton radiotherapy	T3		QLQ-C30, QLQ-OPT30	

### Measures

The EORTC QLQ-C30 questionnaire (version 3.0) was designed to assess general QOL aspects in patients with cancer [18]. Questionnaire responses were evaluated according to the EORTC QLQ-C30 scoring manual (3rd edition).

The EORTC QLQ-OPT30 was designed as an extension module for the QLQ30 and evaluates the QOL in patients with ocular tumors [19]. The questionnaire is focusing on visual and ocular symptoms and consisting of 7 multi-item scales and two additional single items. Responses were evaluated according to the EORTC QLQ-OPT30 scoring manual.

The general anxiety disorder 7 questionnaire (GAD-7) is a validated screening tool for assessing the risk and severity of anxiety disorders [20]. Although not specifically designed for use in the context of cancer, the GAD-7 can be assumed to provide useful information about the general psychological baseline condition of patients undergoing cancer treatments. The GAD-7 consists of 7 items that refer to criteria from the Diagnostic and Statistical Manual of Mental Disorders (DSM-5).

### Statistics

Statistical analyses were performed with R (version 4.0.5). A Friedman overall-test was used to detect significant changes over time. When a significant Friedman-test result was observed, post hoc pairwise comparisons between individual time points were performed with the Wilcoxon signed rank test. Differences between independent subgroups were analyzed with a Mann-Whitney-U-test. The correlation analysis was performed using Spearman’s rank correlation coefficient. Overall *p*-values concerning primary outcomes (QLQ-C30, QLQ-OPT30) were considered significant at a two-sided Bonferroni-corrected α level of 0.003. Secondary endpoints, like group comparisons and correlation analyses, are presented without correction for multiple testing, and the corresponding *p*-values (*p* < = 0.05) are considered exploratory.

To identify independent predictors for QLQ-C30 and QLQ-OPT30 outcomes, all variables were included in an age-, sex- and baseline-adjusted multiple linear regression model with backward selection. Additionally, an interaction term was introduced to account for possible interactions between GAD-7 and sex. When the normality assumption was violated, we performed an adequate power transformation.

In case of significant relevance of the interaction in the final model, we summarized the interactions of sex, GAD7 and all timepoints of a given outcome variable into a multiple mixed linear regression model. Estimated marginal means of the outcome values with 95% confidence intervals (CI) at different time points and in different subgroups are reported and displayed graphically.

Summary tables including all correlation heatmaps, final multiple linear regression models and figures on marginal means are provided as an attachment to this article (Additional files 1, 2, 3, 4, 5, 6, 7, 8, 9, 10, 11, 12, 13, 14, 15, 16, 17, 18, 19, 20, 21, 22.

## Results

### Study sample

Of 183 patients diagnosed with UM that were eligible for proton therapy, 160 patients met the inclusion criteria. We excluded ten patients (5.5%) for insufficient German language skills and seven (3.8%) due to a recurrence treatment. Six patients (3.3%) withdrew consent for proton therapy after the eligibility screening; instead, these patients preferred enucleation (5 patients) or a watch-and-wait strategy (1 patient). Of the 160 patients, 28 (15.3%) could not be contacted before clip-surgery, and therefore, were not included. Only one patient declined participation. Finally, 66 male and 65 female patients (total 131 patients) participated in the prospective questionnaire trial. The mean age of the cohort was 59.12 years (SD ± 13.6), and male and female patients did not differ significantly in age. All tumor characteristics were equally distributed throughout the age and gender subgroups. Detailed information on patient and tumor characteristics are summarized in Table 2. The questionnaire return rates were 90.1% (118 patients) at T0, 99.2% (130 patients) at T1, 91.6% (120 patients) at T2, and 83.1% (108 patients) at T3. The median time interval was 13 days (IQR 14 days) between clip surgery (T0) and T1, 23 days (IQR 20 days) between clip surgery (T0) and T2 and 97 days (IQR 7 days) between proton therapy (T2) and T3.

**Table 2:** Patient and tumor characteristics

Characteristics	All patients	Male	Female	
Patients, n	131	66	65	
Mean age, y (range)	59.1(20–84)	59.8 (30–84)	58.4(20–80)	n.s.
T1a/c	49 (37%)	23 (35%)	26 (40%)	n.s.
T2a/b	45 (34%)	26 (39%)	19 (29%)	n.s.
T3a/b	30 (23%)	15 (23%)	15 (23%)	n.s.
T4a/b	7 (5%)	2 (3%)	5 (8%)	n.s.
*Tumor sub-site*				
Uvea only (without ciliary body involvement)	117 (89.3%)	62 (93.9%)	55 (84.6%)	n.s
Ciliary body only	2 (1.5%)	1 (1.5%)	1 (1.5%)	n.s
Combined ciliary body and uvea	12 (9.2%)	3 (4.5%)	9 (13.8%)	n.s.
Iris only	0 (0%)	0 (0%)	0 (0%)	n.s.
*Basic metrical tumor characteristics*				
Mean tumor prominence (mm)	4.54 ± 3.03	4.42 ± 2.72	4.66 ± 3.34	n.s.
Mean tumor base diameter (mm)	14.99 ± 3.88	14.9 ± 14.95	15.08 ± 3.94	n.s.
Mean tumor volume (mm^3^)	476 ± 444	434 ± 315	519 ± 518	n.s.
Mean distance between tumor and fovea (mm)	1.91 ± 3.04	1.80 ± 2.90	2.02 ± 3.25	n.s.
Mean distance between tumor and optic disc (mm)	2.55 ± 3.25	2.89 ± 3.46	2.20 ± 3.01	n.s.
Adjuvant treatment*				
No adjuvant treatment recommended	68 (51.9 %)	32 (48.5 %)	36 (55.4 %)	n.s
Intervention considered	63(48.1 %)	34 (51.5 %)	29 (44.6 %)	n.s

n.s. = no significant difference between the gender subgroups (Mann-Whitney-U-Test and Chi-square-Test). Tumor staging classes are based on the TNM system of the American Joint Committee on Cancer, 8th edition. Values are the number of patients (%) or the mean ± standard deviation. *Adjuvant treatment procedure as recommended by the pre-treatment tumor boardl

### Quality of life

#### EORTC QLQ-C30

The global QOL did not significantly change between the pretreatment (T0) and the final assessment (T3). However, a significant decline in global QOL was observed after the clip surgery procedure (T1) compared to baseline (T0) (*p* ≤ 0.001). In particular, all functional scales, except “emotional functioning” and “social functioning”, significantly deteriorated after surgery (*p* ≤ 0.001). “Emotional functioning” and “social functioning” remained stable. The most prominent changes occurred in “role functioning”, where the mean index declined from 83.6 to 68.8 (*p* ≤ 0.001). Immediately after completing the proton treatment (T2), the global QOL showed a trend of improvement, which occurred in all functional scales, except “physical functioning”. Three months after treatment (T3), the global QOL returned to baseline status (T0). However, “physical functioning” and “role functioning” remained significantly worse compared to the pre-treatment baseline status (*p* < 0.001). In contrast, “social functioning” and “emotional functioning” gradually improved over time.

#### EORTC QLQ-OPT30

Our patients reported significantly more eye-specific symptoms three months after the treatment (T3) compared to pre-treatment (T0). The symptoms encompassed all multi-item scales (*p* ≤ 0.001), except “worry about recurrence”, which improved significantly over time (*p* ≤ 0.001).

The most prominent decline in QOL appeared after clip surgery (T0-T1). Although “ocular irritation” improved gradually after surgery (T1–T3), the scales “visual impairment”, “problems with exterior aspect”, and “problems with reading” remained unchanged over time. In contrast, “functional problems with the treated eye” and “problems with driving” showed a trend of further deterioration after surgery. More details on the functional scales and single items on the QLQ-C30 and QLQ-OPT30 questionnaires are given in Tables 3 and 4.

**Table 3:** Mean score values for the global QOL and the different functional scales of the EORTC QLQ-C30

EORTCQLQ-C30 Scales	T0	T1	T2	T3	Friedman Test	*p* (T0:T1)	*p* (T1:T2)	*p* (T2:T3)	*p* (T0:T3)
Global QOL	68.22 ± 8.96	63.54 ± 20.2	66.04 ± 20.04	68.75 ± 18.09	**0.001**	**0.001**	0.130	0.41	0.431
*Functional scales*									
Physical functioning	93.01 ± 14.61	89.37 ± 17.28	89.24 ± 16.52	86.38 ± 19.04	**< 0.001**	**< 0.001**	0.796	0.005	**< 0.001**
Role functioning	83.62 ± 25.33	68.85 ± 31.57	73.75 ± 28.39	72.48 ± 25.41	**< 0.001**	**< 0.001**	0.018	0.113	**< 0.001**
Cognitive functioning	87.43 ± 17.42	81.27 ± 19.01	83.89 ± 21.14	81.45 ± 21.12	**< 0.001**	**< 0.001**	0.014	0.069	0.004
Emotional functioning	64.27 ± 26.01	65.35 ± 25.76	68.49 ± 23.32	73.30 ± 21.92	0.088				
Social functioning	81.36 ± 24.57	74.68 ± 26.20	79.55 ± 23.71	83.01 ± 22.51	0.006				

QOL: quality of life; EORTC QLQ-C30: European Organization for Research and Treatment of Cancer QOL questionnaire; T0: before clip-surgery; T1: between clip-surgery and proton therapy; T2: after proton therapy; and T3: three months after treatment. Values are the mean scores ± standard deviation. Bold font: In cases where the Friedman pre-test showed significance, changes between two assessment points are considered significant for *p*-values ≤ 0.003

**Table 4:** Mean Score values for the multi-item QOL scales and single items for the EORTC QLQ-OPT30

EORTC QLQ-OPT30	T0	T1	T2	T3	Friedman Test	*p* (T0:T1)	*p* (T1:T2)	*p* (T2:T3)	*p* (T0:T3)
*Multi-Item-Scales*									
Ocular irritation	12.42 ± 14.73	31.62 ± 18.47	27.55 ± 19.24	22.71 ± 18.99	**< 0.001**	**< 0.001**	**0.001**	0.026	**< 0.001**
Visual impairment	15.33 ± 18.51	24.46 ± 23.87	21.84 ± 24.67	22.85 ± 23.25	**< 0.001**	**< 0.001**	0.158	0.272	**< 0.001**
Worry about recurrence	61.68 ± 29.65	56.88 ± 29.79	48.81 ± 30.82	46.18 ± 28.59	**< 0.001**	0.031	**< 0.001**	0.815	**< 0.001**
Problems with exterior aspect	6.32 ± 18.08	23.71 ± 27.05	25.68 ± 27.13	21.86 ± 25.96	**< 0.001**	**< 0.001**	0.718	0.464	**< 0.001**
Functional problems with visual impairment	13.32 ± 19.38	22.67 ± 22.56	22.92 ± 23.92	26.00 ± 22.6	**< 0.001**	**< 0.001**	0.954	0.010	**< 0.001**
Functional problems with treated eye	25.64 ± 21.48	29.18 ± 22.37	30.83 ± 23.39	33.11 ± 22.18	**< 0.001**	**0.001**	0.952	0.157	**< 0.001**
Problems with driving	21.52 ± 24.99	26.15 ± 30.71	26.64 ± 29.13	35.78 ± 30.7	**< 0.001**	0.009	0.633	0.030	**< 0.001**
*Single Items*									
Headache	14.97 ± 24.50	16.15 ± 24.64	16.24 ± 26.12	20.44 ± 29.3	0.356				
Problems with reading	33.33 ± 35.83	41.93 ± 30.24	40.62 ± 29.8	42.68 ± 29.24	0.010				

QOL: quality of life; EORTC QLQ-OPT30: European Organization for Research and Treatment of Cancer QOL questionnaire-ophthalmic oncology module; T0: before clip-surgery; T1: between clip-surgery and proton therapy; T2: after proton therapy; and T3: three months after treatment; Bold font: In cases where the Friedman pre-test showed significance, changes between two assessment points are considered significant for *p*-values ≤ 0.003

#### EORTC QLQ-C30 and patient sex

The significant decline in global QOL between pretreatment (T0) and the post-surgical assessment (T1) was only observed among female patients (*p* ≤ 0.001). Before (T1) and directly after proton therapy (T2), the overall QOL was significantly lower among female patients than among male patients (T1: *p* = 0.016, T2: *p* = 0.042). Global QOL in female patients showed a trend of improvement immediately after proton therapy (T2), then returned to baseline values three months after proton therapy (T3). Interestingly, male patients did not show any significant QOL changes during the treatment process. All functional scales, except “physical functioning“, remained stable among male patients over the entire study period (T0-T3). Female patients showed an additional decline in “role functioning” and “cognitive functioning”, between pretreatment (T0) and the post-surgical assessment (T1) (*p* ≤ 0.001). Although we observed a partial recovery of these functional scales in female patients over time, “physical functioning”, “role functioning”, and “cognitive functioning” remained significantly reduced at 3 months after treatment (T3), compared to pretreatment values (T0) (*p* ≤ 0.003). In general, female patients showed significantly lower “emotional functioning” than male patients at all assessment points (T0: *p* = 0.042, T1: *p* = 0.037, T2: *p* = 0.014, T3: *p* = 0.004).

The multiple linear regression analysis identified significant associations between patient sex and “cognitive functioning” at T0 (*p* = 0.023), “role functioning” at T0 and T1 (*p* ≤ 0.032) and “physical functioning” at T2 (*p* = 0.014).

#### EORTC QLQ-OPT30 and patient sex

Before treatment (T0), we observed no significant differences between male and female patients in any of the multi-item scales of the EORTC QLQ-OPT30 questionnaire. However, at later assessments (T1–T3), female patients reported more severe impairments in all multi-item-scales and single-items, compared to male patients. Significantly worse scores in “visual impairment”, “functional problems with the treated eye”, and the single item, “difficulties with reading”, were only observed among female patients during the therapy process.

For both sexes, “worry about recurrence” improved significantly over time (T0–T3) (*p* ≤ 0.002), although women were significantly more affected than men at 3 months after treatment (T3) (*p* ≤ 0.01).

The multiple linear regression analyses showed that female sex was an independent predictor of the single-item “headache” at T2 (*p* = 0.006).

#### GAD-7 and patient sex

The GAD-7 was completed once by patients before proton therapy started (T1). The questionnaire return rate was 98.5%. The mean GAD-7 score was 5.91 (SD ± 4.28). Fifty-four patients (41.9%) showed no signs of generalized anxiety, 54 (41.9%) patients presented mild anxiety, 16 (12.4%) patients showed intermediate anxiety, and 5 (3.9%) patients showed severe signs of anxiety.

Before proton therapy (T1), signs of general anxiety were reported more frequently by female patients than by male patients. The majority of women (72.3 %) showed at least mild signs of anxiety. Male patients had a significantly lower mean GAD-7 sum score compared to female patients (*p* ≤ 0.001). The multiple linear regression analysis revealed that female sex was the only significant predictor of a worse GAD-7 outcome (*p* < 0.001).

#### Age and tumor characteristics

The multiple linear regression analysis showed no significant associations between any tumor characteristic and any QLQ-C30 subscale at any time. In contrast, tumor prominence was identified as a significant predictor for several multi-items of the QLQ-OPT30: “ocular irritation” (T3), “visual impairment” (T0, T3), “functional problems with visual impairment” (T0, T3), “functional problems with treated eye” (T0), “problems with driving” (T3), “worry about recurrence” (T1) (*p* ≤ 0.001–0.049). In addition, smaller distance between tumor and fovea was a significant predictor for “problems with reading” (T1), “visual impairment” (T0), “functional problems with visual impairment” (T0) and “functional problems with treated eye” (T0) (*p* ≤ 0.013–0.037).

In univariate analyses, age was not significantly correlated with the baseline global QOL outcome. However, the multivariate analyses revealed that a younger age was a significant predictor for a worse outcome in “global health“ (T1; *p* = 0.021), “emotional functioning” (T0; *p* < 0.029), “role functioning” (T2; *p* = 0.048), “ocular irritation” (T1; *p* = 0.019) and “headache” (T0, T3; *p* ≤ 0.04). In contrast, older age was associated with worse “visual impairment” (T2; *p* = 0.019), “functional problems with visual impairment” (T2; *p* = 0.031) and “worry about recurrence” (T2; *p* = 0.025).

#### GAD-7 and QOL

We found that general anxiety was a crucial factor for QOL issues in our patients. Univariate analyses showed significant correlations between the mean GAD-7 score and QOL outcome in all functional scales (except “role functioning“ at T0) and every eye-related impairment, at all assessment points (T0-T3).

Our multiple linear regression results showed that the GAD-7 was associated with worse QOL measures for all the multi-items on the QLQ-OPT30 at baseline (except “visual impairment”) (p ≤ 0.013) and before proton treatment (T1; *p* = 0.016). Moreover, after proton treatment (T2), the GAD-7 could significantly predict a worse QOL outcome with respect to all multi-items of the QLQ-OPT30 (p ≤ 0.047), except for “headache”, “problems with driving”, “problems with exterior aspect” and “functional problems with treated eye”. Additionally, the GAD-7 was significantly associated with a worse QOL outcome based on all the subscales of the QLQ-C30 at all timepoints (*p* ≤ 0.001–0.042), except for the global QOL at T2, “emotional functioning” at T2 and “physical functioning” at T1 and T2.

### Interaction of GAD7 and sex

Interaction analyses revealed relevant interactions between the predictors sex and GAD7 with respect to the outcome of “emotional functioning”, “problems with exterior aspect”, “ocular irritation” and “global QOL”. Female patients with higher GAD7 sum score were more likely to experience treatment related burden compared to male patients.

## Discussion

The current study represented the first prospective, systematic evaluation of patient reported outcome measures in a large group of patients with UM that were treated with proton therapy. To date, no previous studies have investigated in detail QOL issues of patients with UM at different stages of the proton treatment. To discriminate between interventional and non-interventional issues that contributed to the overall outcome, we collected additional QOL data between the clip surgery and the proton therapy, and then, again after completing the proton therapy. With the exception of “emotional” and “social functioning”, all the subscales of the QLQ-C30 and all the eye-related multi-items of the QLQ-OPT30 were significantly worse after the clip surgery and before the beginning of proton therapy (T1) in female patients. There was a trend of recuperation in most QLQ-C30 and QLQ-OPT30 scales at the completion of proton therapy, compared to the post-surgical assessment. Therefore, the overall impact of the surgical part of the treatment on QOL seemed to be crucial to the general treatment burden. This result emphasized the need for reliable, non-invasive alternative methods for positioning patients during proton therapy. To that end, Via et al. and others have been developing eye-tracking-based methods to obviate clip surgery [21–24]. Moreover, for selected patients with larger UM tumors, alternative approaches might be available that are entirely non-invasive, like frameless stereotactic radiotherapy or Cyberknife treatment, which have acceptable QOL outcomes [25, 26].

The majority of previous studies have investigated long-term QOL issues in patients with UM that underwent different treatment strategies. Only a few studies have focused on the short-term treatment-related impact of primary UM therapy [7, 10, 27, 28]. Our results from the baseline assessments (T0) were generally consistent with previous studies that reported pre-treatment data [7, 9]. In our cohort, the pre-treatment status, measured with the global QOL and all the functional subscales, except for “social functioning”, was similar to findings by Barker et al., Suchocka-Capuano et al., and van Beek et al.[7, 9, 10] (see also Table 5). The more severe effects on “social functioning” observed in our cohort may reflect the national characteristics observed in the QLQ-C30 for the German norm population, as described by Nolte et al. [29]. Three months after therapy, the global QOL of our patients was stable compared to the baseline assessment. This result was consistent with results from earlier studies on the QOL of patients with UM [7, 10].

**Table 5:** Mean pretreatment score values for global QOL and functional subscales compared between the present study and previous studies with available pretreatment data on patients with UM

EORTC QLQ-C30 scales	Present study, n = 118	Barker et al. (2020), n = 200	Suchocka-Capuano et al. (2011), n = 69	Van Beek et al. (2018), n = 65
Global QOL	68.22 ± 08.96	76.1 ± 21.7	68.8 ± 19.3	76.4 ± 13.6
Physical functioning	93.01 ± 14.61	91.5 ± 17.0	89.7 ± 15.1	85.9 ± 19.6
Role functioning	83.62 ± 25.33	90.4 ± 21.5	82.4 ± 25.4	80.0 ± 29.1
Emotional functioning	64.27 ± 26.01	78.1 ± 20.2	75.4 ± 21.2	69.0 ± 22.7
Cognitive functioning	87.43 ± 17.42	87.8 ± 18.5	83.6 ± 18.6	80.8 ± 21.5
Social functioning	81.36 ± 24.57	90.6 ± 20.1	93.7 ± 15.2	92.6 ± 12.5

QOL: quality of life; UM: uveal melanoma; EORTC QLQ-C30: European Organization for Research and Treatment of Cancer QOL questionnaire

A direct comparison with existing literature was limited, for several reasons. First, previous studies included different treatment strategies; second, data acquisition was inhomogeneous, with respect to the follow-up strategy; and third, some selected different questionnaires. For example, Hope-Stone et al. did not report QLQ-C30 data, but showed similar results regarding “ocular irritation” and “worry about recurrence” at 6 months after treatment [13, 14]. However, in that study, no pre-treatment assessment data were available to evaluate treatment-related changes over time. Moreover, a functional adaption to visual impairments was assumed to play a major role in the early months after treatment [30]. In another study, Suchocka-Capuano et al. provided pre-treatment and one-month follow-up data on 69 patients with UM [7]. Those authors described similar ocular and visual outcomes compared to our study for the subscales “functional problems due to visual impairment”, “problems with reading”, and “problems with driving”. In contrast to our results, Suchocka-Capuano et al. reported no significant changes between the baseline assessment and the one-month follow-up, regarding the multi-item scales, “ocular irritation”, “visual impairment” and “worry about recurrence” [7].

We next compared the pre-treatment QLQ-C30 findings from our cohort with general population norm data for Germany [29]. Our patients showed pre-treatment scores similar to those of the norm, for the following items: the global QOL (68.2 vs. 67.0), “role functioning” (83.6 vs. 80.8), “cognitive functioning” (87.4 vs. 83.9), and “social functioning” (81.4 vs. 84.8). However, our patients reported better scores for “physical functioning” (93.0 vs. 82.8) and worse scores for “emotional functioning” (64.3 vs. 73.9), compared to the norm data. The “emotional functioning“ subscale was previously associated with anxiety and depression in patients with cancer [31]. We found that worse “emotional functioning” at baseline (T0) was correlated with a high proportion of patients with anxiety issues, according to the GAD-7. Moreover, worse “emotional functioning” corresponded to an elevated pre-treatment “worry about recurrence”, as previously described by Barker et al. [9]. In our study, “emotional functioning” subsequently improved after treatment, which was consistent with findings in previous studies [7, 10, 27, 32]. Finally, we found that 3 months after treatment, “emotional functioning” returned to standard values for the German population [29].

Interestingly, in our study, the “worry about recurrence” improved significantly from T0 to T1, although proton therapy had not yet started. A potential explanation for this result may be that “waiting for the treatment” was a burden to many patients. Once a patient initiated the treatment preparation process, fighting cancer had begun. This attitude might have had a positive impact on the patient’s mental condition, despite the fact that the most relevant part of the treatment was pending.

For our patients, the “worry about recurrence” continued to improve until they completed radiotherapy (T2); then, it remained stable for 3 months (T3). Previous studies on patients with UM described a relief from anxiety during the long-term follow-up [7, 10, 27]. Our data suggested that the observed effect may be more closely related to the beginning and completion of the treatment procedure, rather than reflecting the long-term evolution.

In our study, female patients showed a significantly lower “emotional functioning” and a higher level of “worry about recurrence”, than male patients in univariate analyses. These results were also described in several previous studies [9, 13, 28]. Moreover, we found a significantly higher GAD-7 sum score in female patients than in male patients, as previously reported for women in the German norm population [20]. It is known that, generally, after a cancer diagnosis, increased anxiety symptoms are more frequently reported by women than by men [33].

Interestingly, we observed that the female sex was significantly correlated with the declines in global QOL and ocular symptom scores after the clip surgery. However, we observed no differences in global QOL between the sexes at baseline. A potential explanation may be that, in our study, the female patients reported a higher level of anxiety, compared to the male patients. Indeed, the level of anxiety might have affected how patients experienced even clearly somatic symptoms. This interpretation was confirmed by identifying the GAD-7 sum score as the most relevant predictor for QOL outcome in multiple linear regression analyses at all time-points in our study. In addition, female patients reporting high anxiety levels showed increased treatment-related symptoms compared to male patients.

The main strength of our study was the detailed exploration of health issues closely related to treatment reflected in the short-term QOL measured before and after both, proton therapy and the associated clip surgery. We observed high levels of overall compliance and questionnaire return rates. These findings might have reflected the need for mental support by patients during the treatment procedure.

This study had several limitations. The long-term, radiation-induced toxicity in patients with UM tends to evolve gradually, over months and years, after proton therapy. Therefore, our data did not allow comparisons to existing long-term QOL data on patients with UM. We plan to address the long-term QOL in our patients in the future. On the other hand, we may have missed early, transient radiation-related side-effects, which are expected to peak at one to three weeks after the end of proton therapy. Another limitation was that we only focused on the side-effects of the proton therapy procedure; we did not take into account the impact of possible adjuvant treatments on the QOL of our patients.

In conclusion, to our knowledge, this study was the first to explore, prospectively and in detail, the treatment-related burden imposed during the different stages of proton therapy in a homogeneously treated cohort of patients with UM. Our finding that the QOL in these patients was more severely affected by the clip surgery than by the radiation procedure suggesting that future efforts should focus on establishing alternative non-invasive methods for positioning patients during ocular proton therapy. We also found that a higher level of anxiety was a significant predictor for QOL outcome. Psycho-oncologic patient care should address these issues early in the treatment process to improve treatment acceptability and QOL outcomes.

## Supplementary Information

**Additional file 1**. Heatmap showing spearman’s rank correlation coefficients between all variables regrading a given subscale for timepoints T0-T3.

**Additional file 2**. Heatmap showing spearman’s rank correlation coefficients between all variables regrading a given subscale for timepoints T0-T3.

**Additional file 3**. Heatmap showing spearman’s rank correlation coefficients between all variables regrading a given subscale for timepoints T0-T3.

**Additional file 4**. Heatmap showing spearman’s rank correlation coefficients between all variables regrading a given subscale for timepoints T0-T3.

**Additional file 5**. Heatmap showing spearman’s rank correlation coefficients between all variables regrading a given subscale for timepoints T0-T3.

**Additional file 6**. Heatmap showing spearman’s rank correlation coefficients between all variables regrading a given subscale for timepoints T0-T3.

**Additional file 7**. Heatmap showing spearman’s rank correlation coefficients between all variables regrading a given subscale for timepoints T0-T3.

**Additional file 8**. Heatmap showing spearman’s rank correlation coefficients between all variables regrading a given subscale for timepoints T0-T3.

**Additional file 9**. Heatmap showing spearman’s rank correlation coefficients between all variables regrading a given subscale for timepoints T0-T3.

**Additional file 10**. Heatmap showing spearman’s rank correlation coefficients between all variables regrading a given subscale for timepoints T0-T3.

**Additional file 11**. Heatmap showing spearman’s rank correlation coefficients between all variables regrading a given subscale for timepoints T0-T3.

**Additional file 12**. Heatmap showing spearman’s rank correlation coefficients between all variables regrading a given subscale for timepoints T0-T3.

**Additional file 13**. Heatmap showing spearman’s rank correlation coefficients between all variables regrading a given subscale for timepoints T0-T3.

**Additional file 14**. Heatmap showing spearman’s rank correlation coefficients between all variables regrading a given subscale for timepoints T0-T3.

**Additional file 15**. Heatmap showing spearman’s rank correlation coefficients between all variables regrading a given subscale for timepoints T0-T3.

**Additional file 16**. Final multiple linear regression models for all endpoints of QLQ-C30 and QLQ-OPT30.

**Additional file 17**. Final mixed linear regression models including all timepoints T0-T3 and an interaction term for GAD-7 and sex for selected endpoints of QLQ-C30 and QLQ-OPT30.

**Additional file 18**. Contrast analyses of estimated marginal means between male and female patients by GAD-7 category for selected endpoints of QLQ-C30 and QLQ-OPT30.

**Additional file 19**. Visualization of marginal estimates of outcome values with 95% confidence interval at different time points (T0-T3) by sex and GAD-7 subcategory.

**Additional file 20**. Visualization of marginal estimates of outcome values with 95% confidence interval at different time points (T0-T3) by sex and GAD-7 subcategory.

**Additional file 21**. Visualization of marginal estimates of outcome values with 95% confidence interval at different time points (T0-T3) by sex and GAD-7 subcategory.

**Additional file 22**. Visualization of marginal estimates of outcome values with 95% confidence interval at different time points (T0-T3) by sex and GAD-7 subcategory.

## Data Availability

The datasets used and analyzed during the current study are available from the corresponding author on reasonable request.

## Abbreviations

UM: Uveal melanoma
QOL: Quality of Life
EORTC QLQ-C30: European Organization for Research and Treatment of Cancer Quality of life questionnaire
EORTC QLQ-OPT30: European Organization for Research and Treatment of Cancer Quality of life questionnaire-ophthalmic oncology module
GAD-7: The general anxiety disorder 7 questionnaire
