# Supplementary material for: Quality of life and treatment-related burden during ocular proton therapy: a prospective trial of 131 patients with uveal melanoma
Source: Radiat Oncol. 2021 Sep 8;16:174. doi: 10.1186/s13014-021-01902-6 (PMC8425039; doi:10.1186/s13014-021-01902-6)
Supplement: Supplementary file 1 — Additional file 1. Heatmap showing spearman’s rank correlation coefficients between all variables regrading a given subscale for timepoints T0-T3. [file 13014_2021_1902_MOESM1_ESM.docx]

**Emotional functioning (EF)**

gad7_cat = GAD7 low (0-4):

contrast estimate SE df t.ratio p.value

male - female -3.04 4.81 119 -0.632 0.5286

gad7_cat = GAD7 middle (5-9):

contrast estimate SE df t.ratio p.value

male - female 5.73 4.71 124 1.216 0.2263

gad7_cat = GAD7 high (10+):

contrast estimate SE df t.ratio p.value

male - female 18.30 7.80 125 2.347 0.0205

**Ocular irritation (OI)**

gad7_cat = GAD7 low (0-4):

contrast estimate SE df t.ratio p.value

male - female -0.556 3.33 118 -0.167 0.8678

gad7_cat = GAD7 middle (5-9):

contrast estimate SE df t.ratio p.value

male - female -0.642 3.28 124 -0.196 0.8450

gad7_cat = GAD7 high (10+):

contrast estimate SE df t.ratio p.value

male - female -16.871 5.43 126 -3.105 0.0023

**Problems with exterior aspect (PA)**

gad7_cat = GAD7 low (0-4):

contrast estimate SE df t.ratio p.value

male - female 0.175 4.8 118 0.037 0.9709

gad7_cat = GAD7 middle (5-9):

contrast estimate SE df t.ratio p.value

male - female -5.731 4.7 123 -1.219 0.2251

gad7_cat = GAD7 high (10+):

contrast estimate SE df t.ratio p.value

male - female -19.019 7.9 130 -2.408 0.0174

**Global health (GH)**

gad7_cat = GAD7 low (0-4):

contrast estimate SE df t.ratio p.value

male - female -0.914 3.83 118 -0.239 0.8118

gad7_cat = GAD7 middle (5-9):

contrast estimate SE df t.ratio p.value

male - female -0.835 3.77 125 -0.222 0.8250

gad7_cat = GAD7 high (10+):

contrast estimate SE df t.ratio p.value

male - female 9.431 6.23 125 1.514 0.1325
