# Supplementary figures and images for: Quality of life and treatment-related burden during ocular proton therapy: a prospective trial of 131 patients with uveal melanoma
Source: Radiat Oncol. 2021 Sep 8;16:174. doi: 10.1186/s13014-021-01902-6 (PMC8425039; doi:10.1186/s13014-021-01902-6)

Heatmap Cognitive functioning T0–T3 (CF)

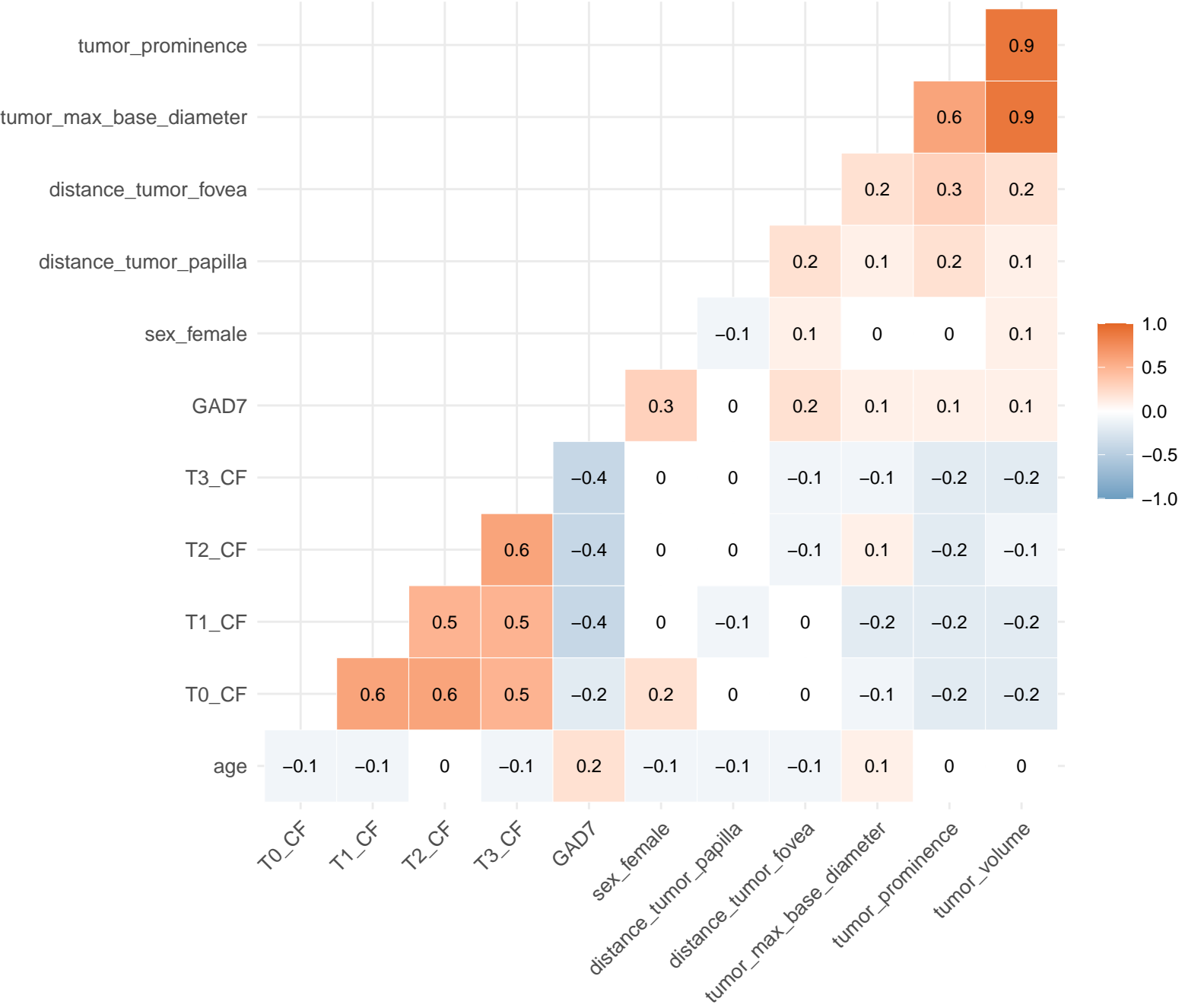

Supplement: Supplementary file 2 — Additional file 2. Heatmap showing spearman’s rank correlation coefficients between all variables regrading a given subscale for timepoints T0-T3. [file 13014_2021_1902_MOESM2_ESM.pdf]

Heatmap Emotional functioning T0–T3 (EF)

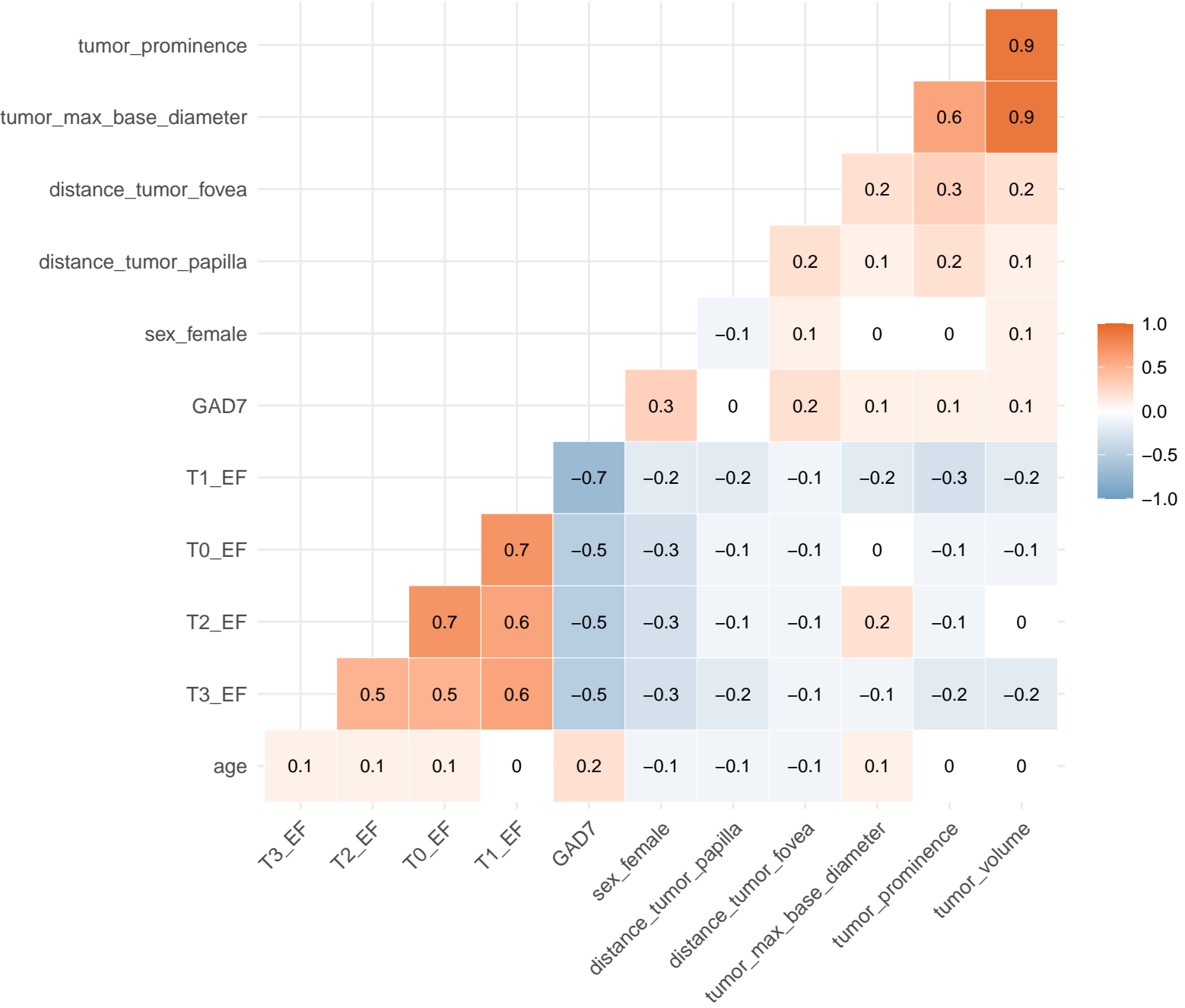

Supplement: Supplementary file 3 — Additional file 3. Heatmap showing spearman’s rank correlation coefficients between all variables regrading a given subscale for timepoints T0-T3. [file 13014_2021_1902_MOESM3_ESM.pdf]

Heatmap Functional problems with treated eye T0–T3 (FPTE)

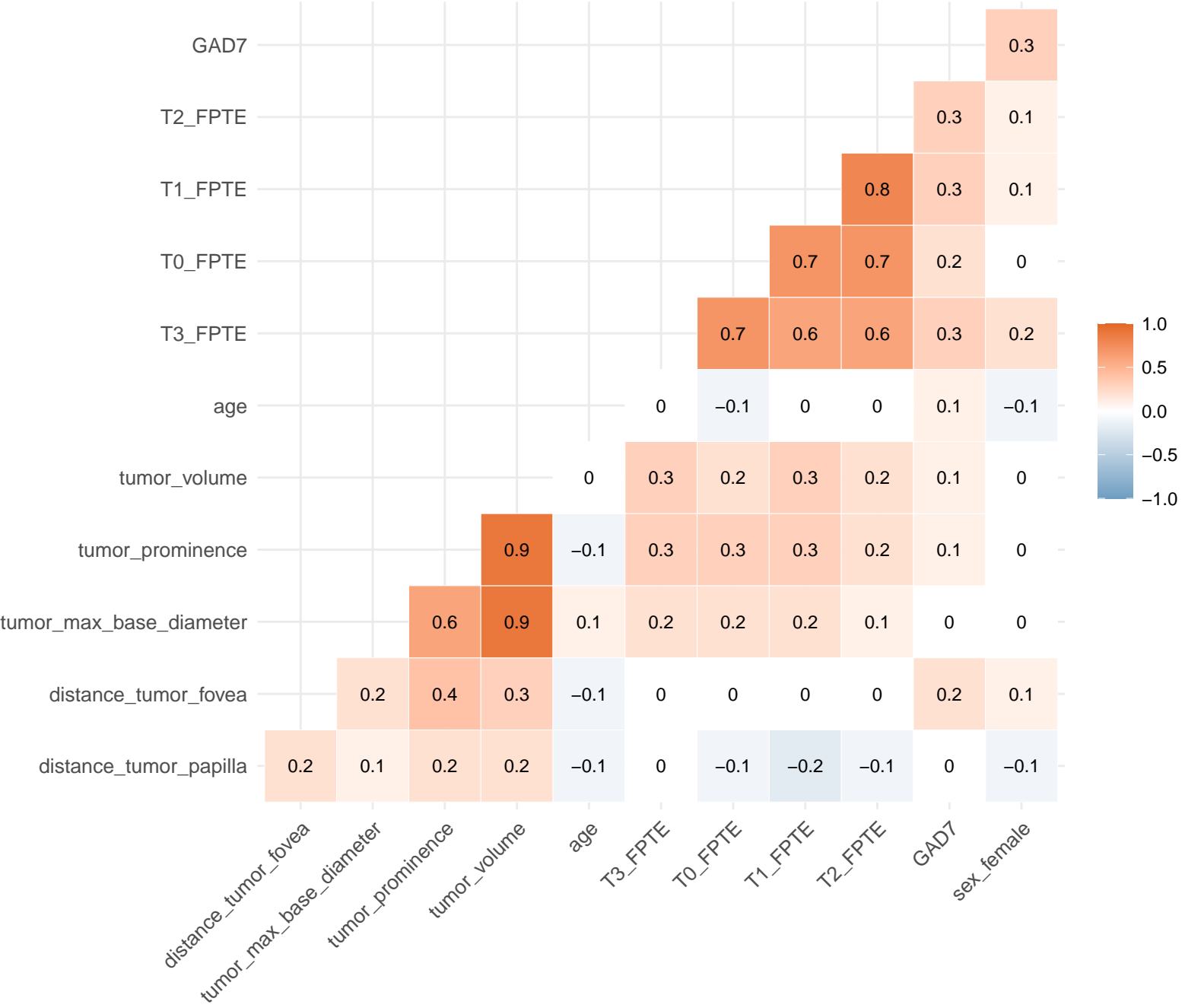

Supplement: Supplementary file 4 — Additional file 4. Heatmap showing spearman’s rank correlation coefficients between all variables regrading a given subscale for timepoints T0-T3. [file 13014_2021_1902_MOESM4_ESM.pdf]

Heatmap Functional problems with visual impairment T0–T3 (FPVI)

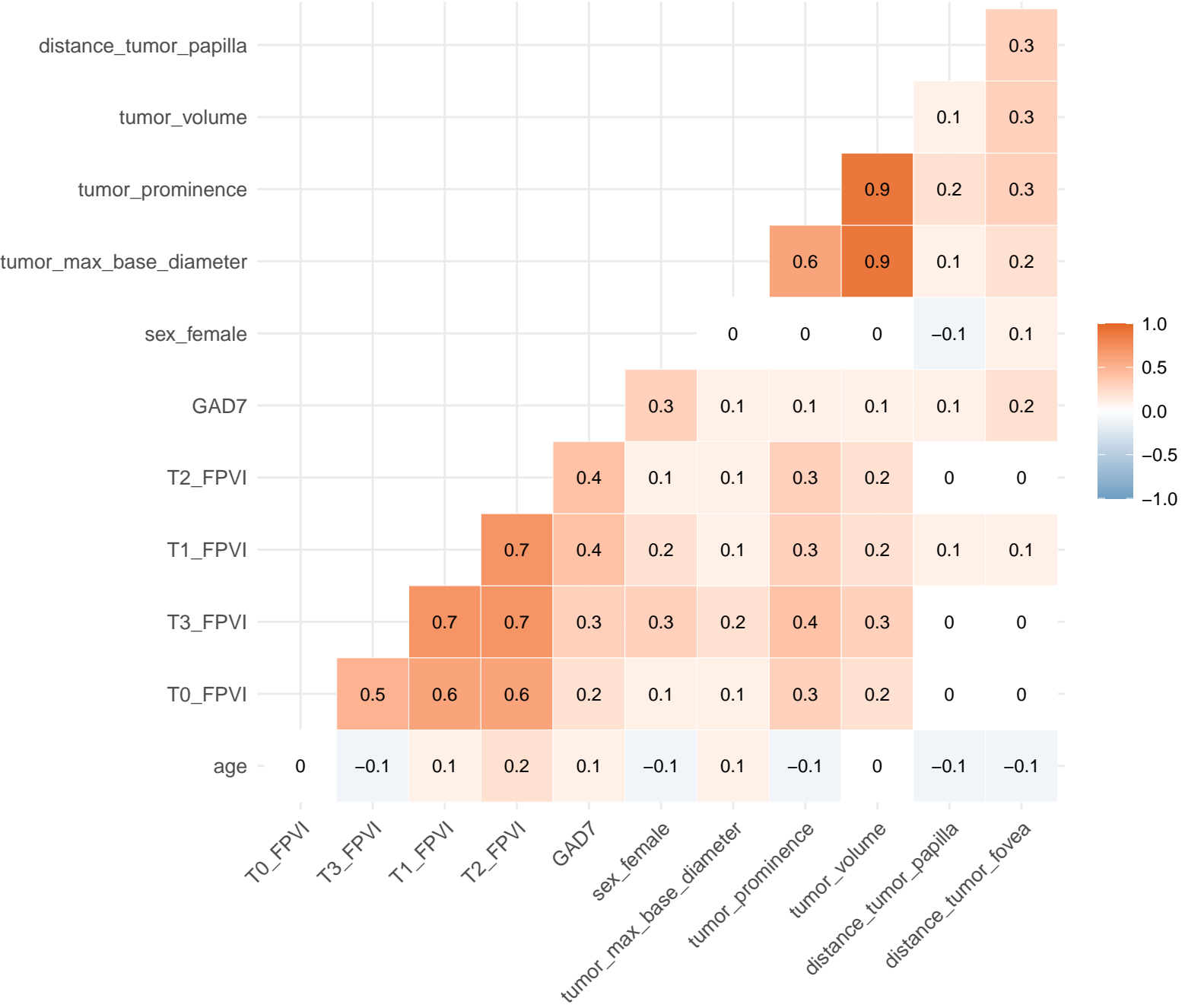

Supplement: Supplementary file 5 — Additional file 5. Heatmap showing spearman’s rank correlation coefficients between all variables regrading a given subscale for timepoints T0-T3. [file 13014_2021_1902_MOESM5_ESM.pdf]

Heatmap Global health T0–T3 (GH)

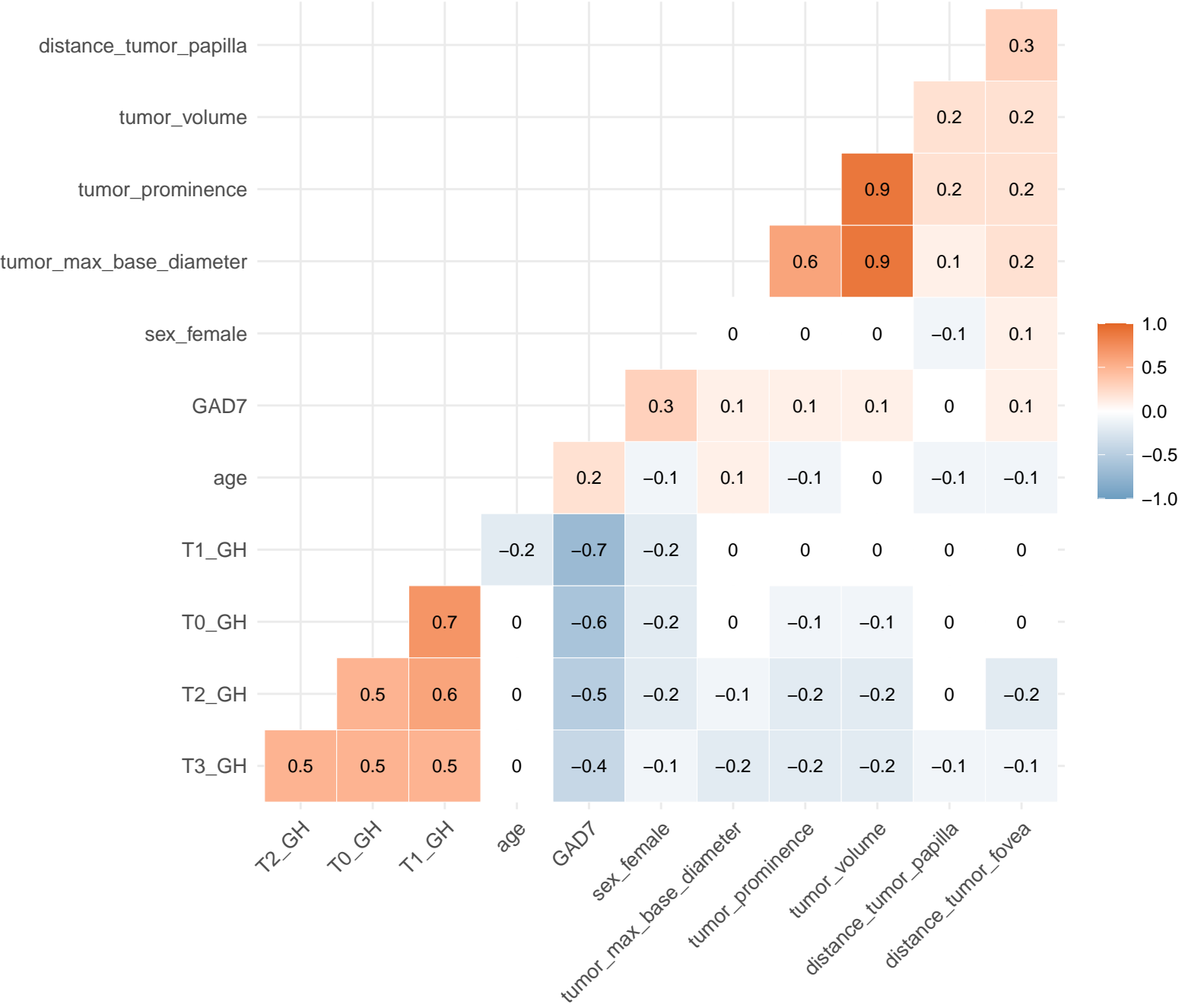

Supplement: Supplementary file 6 — Additional file 6. Heatmap showing spearman’s rank correlation coefficients between all variables regrading a given subscale for timepoints T0-T3. [file 13014_2021_1902_MOESM6_ESM.pdf]

Heatmap Headache T0–T3 (Head)

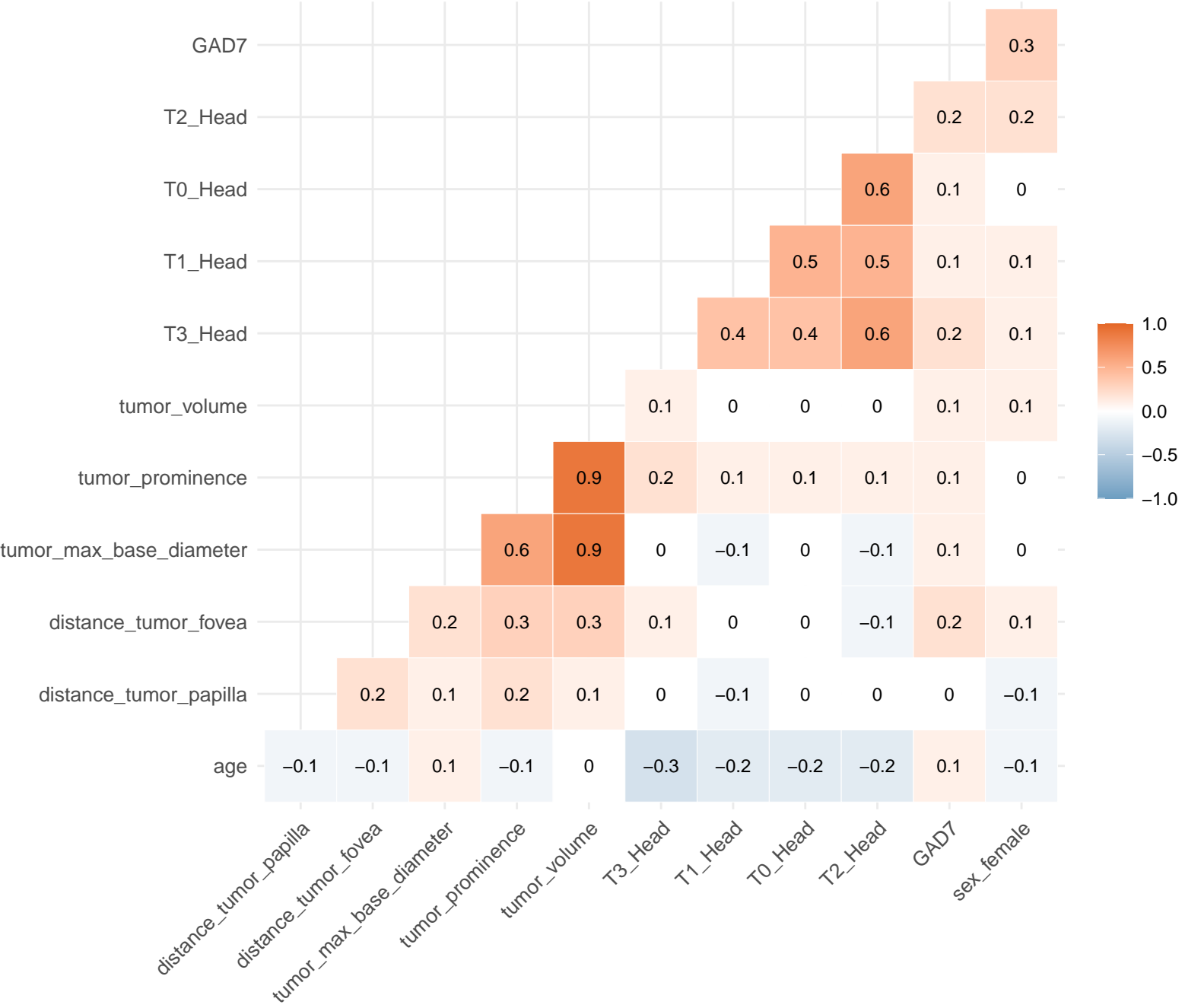

Supplement: Supplementary file 7 — Additional file 7. Heatmap showing spearman’s rank correlation coefficients between all variables regrading a given subscale for timepoints T0-T3. [file 13014_2021_1902_MOESM7_ESM.pdf]

Heatmap Ocular irritation T0–T3 (OI)

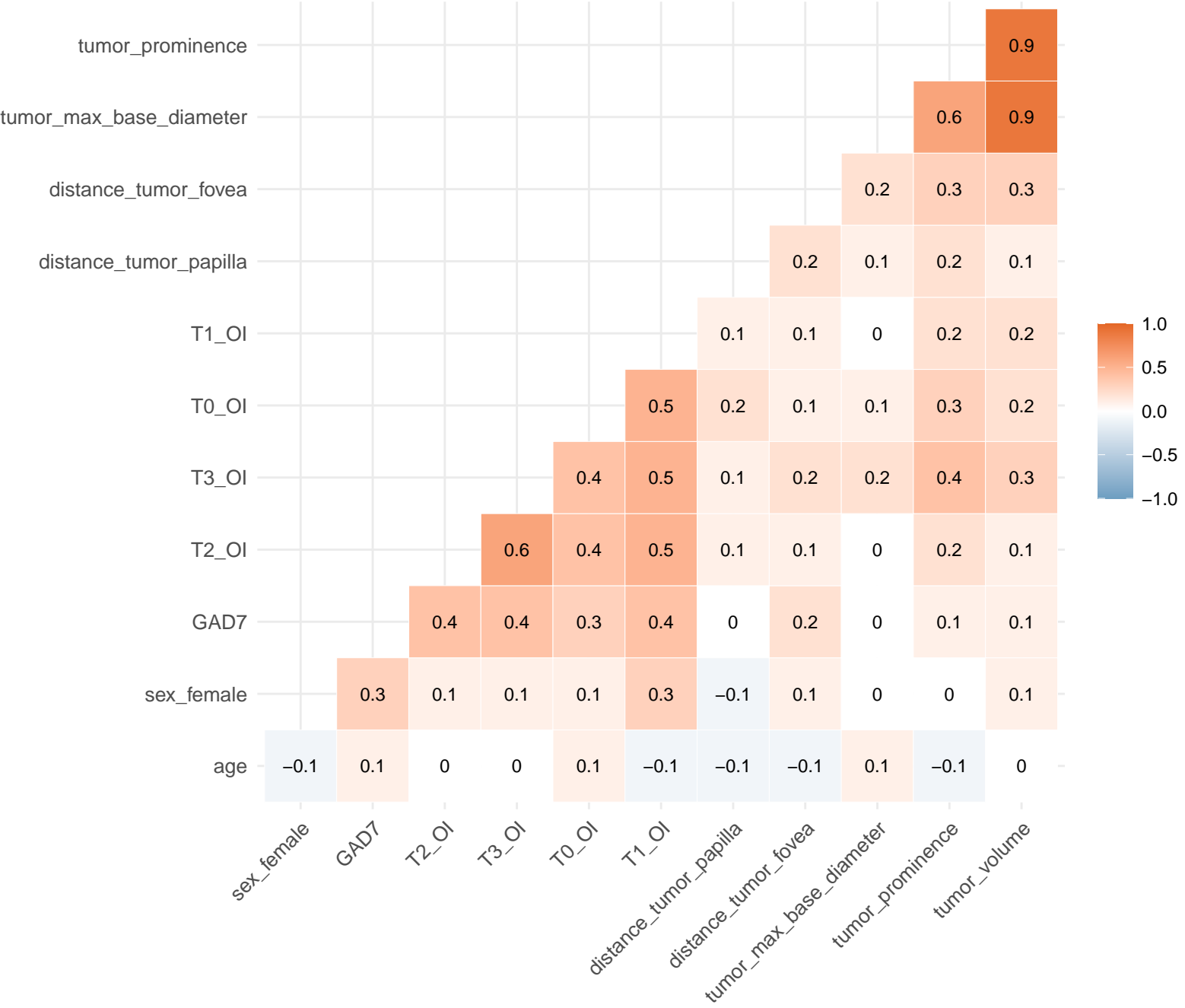

Supplement: Supplementary file 8 — Additional file 8. Heatmap showing spearman’s rank correlation coefficients between all variables regrading a given subscale for timepoints T0-T3. [file 13014_2021_1902_MOESM8_ESM.pdf]

Heatmap Problems with exterior aspect T0–T3 (PA)

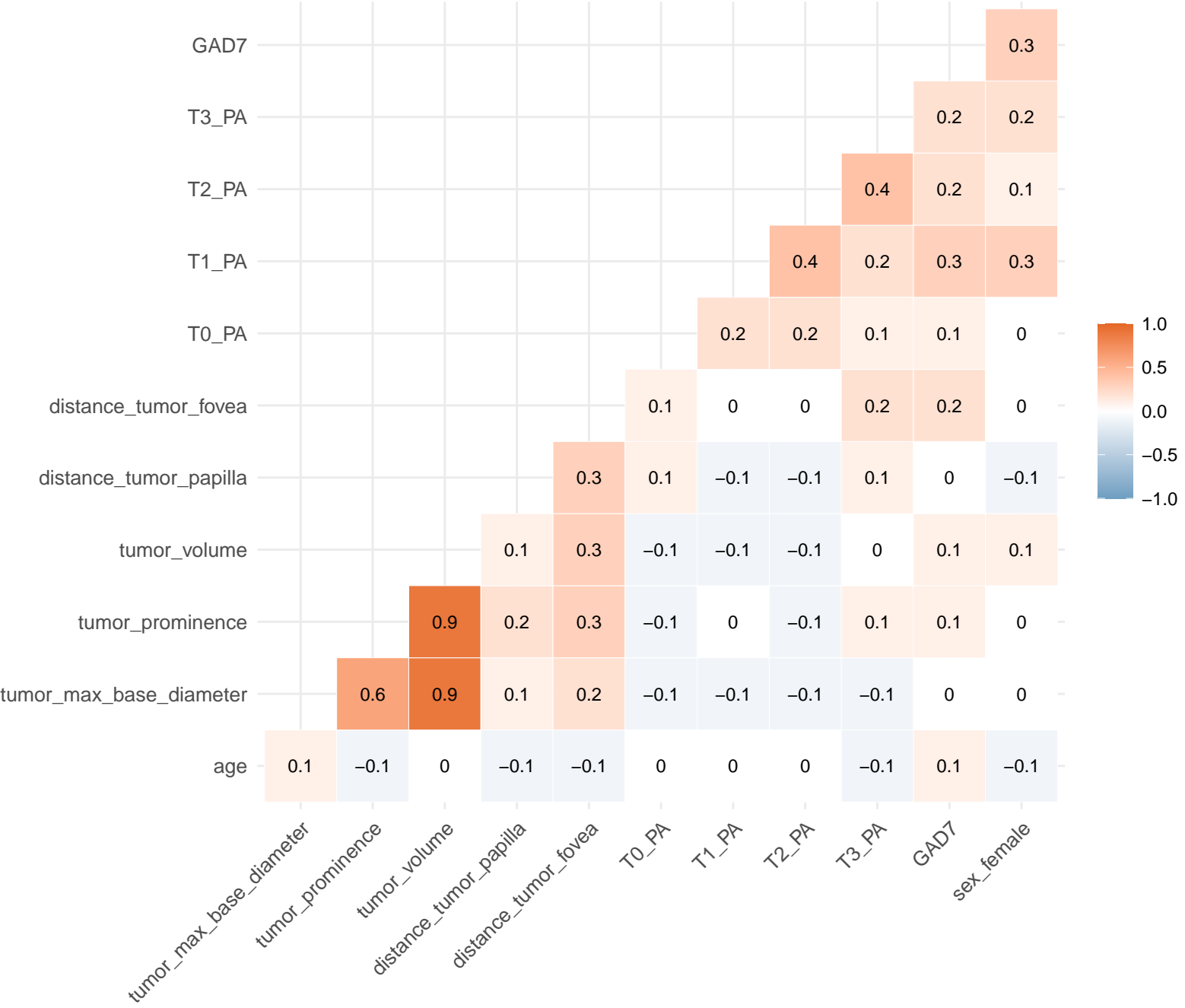

Supplement: Supplementary file 9 — Additional file 9. Heatmap showing spearman’s rank correlation coefficients between all variables regrading a given subscale for timepoints T0-T3. [file 13014_2021_1902_MOESM9_ESM.pdf]

Heatmap Problems with driving T0–T3 (PD)

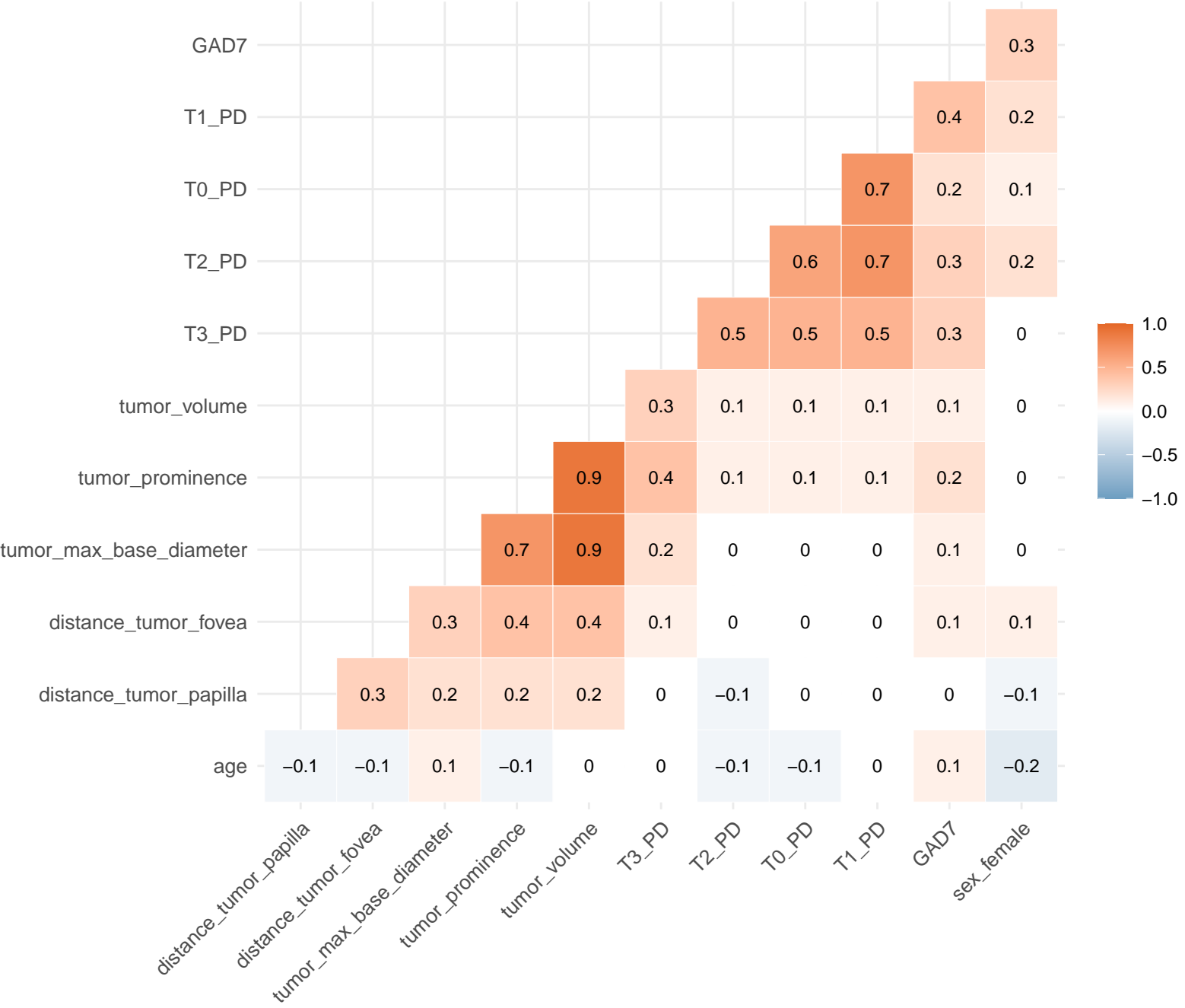

Supplement: Supplementary file 10 — Additional file 10. Heatmap showing spearman’s rank correlation coefficients between all variables regrading a given subscale for timepoints T0-T3. [file 13014_2021_1902_MOESM10_ESM.pdf]

Heatmap Physical functioning T0–T3 (PF)

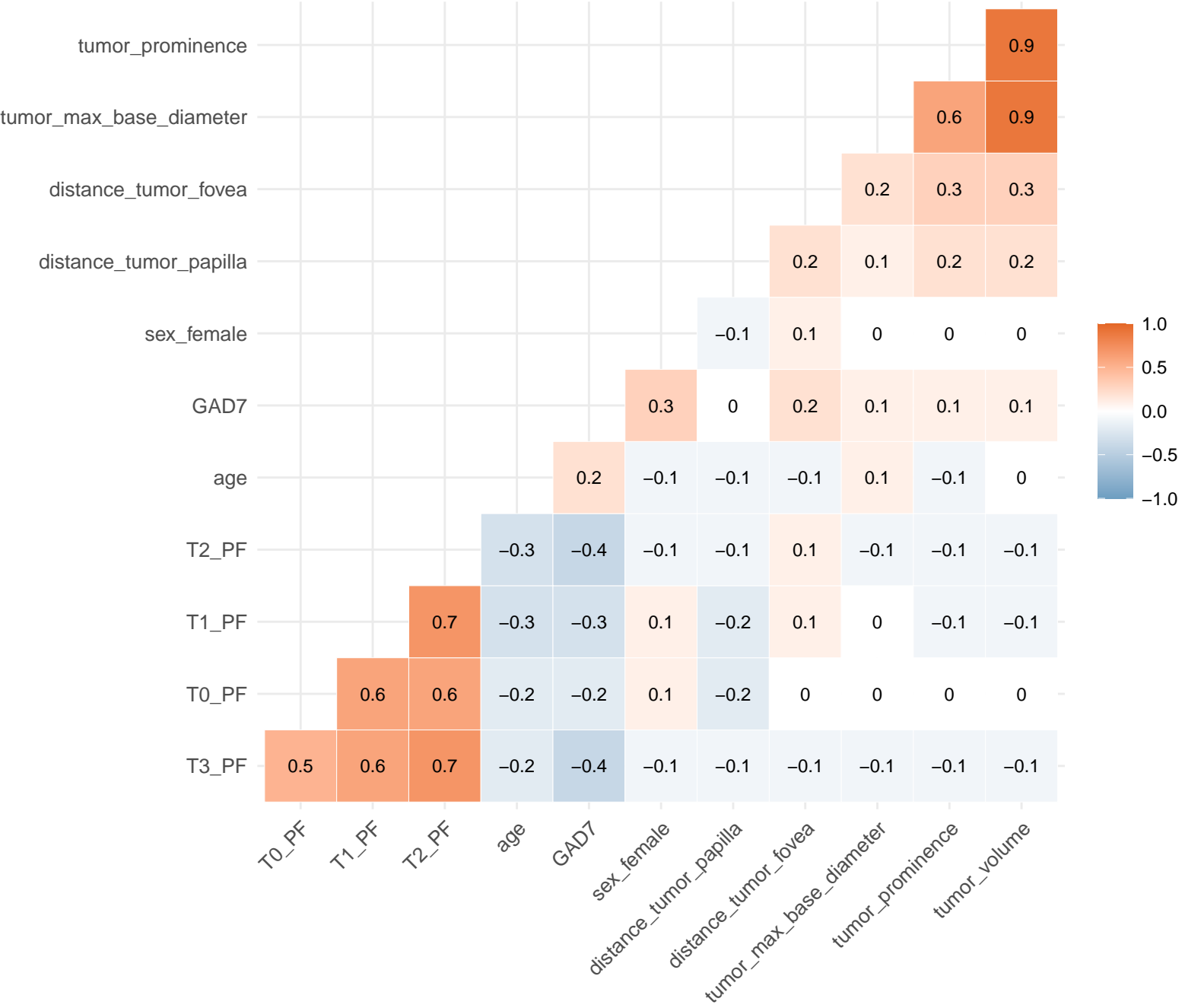

Supplement: Supplementary file 11 — Additional file 11. Heatmap showing spearman’s rank correlation coefficients between all variables regrading a given subscale for timepoints T0-T3. [file 13014_2021_1902_MOESM11_ESM.pdf]

Heatmap Problems with reading T0–T3 (PR)

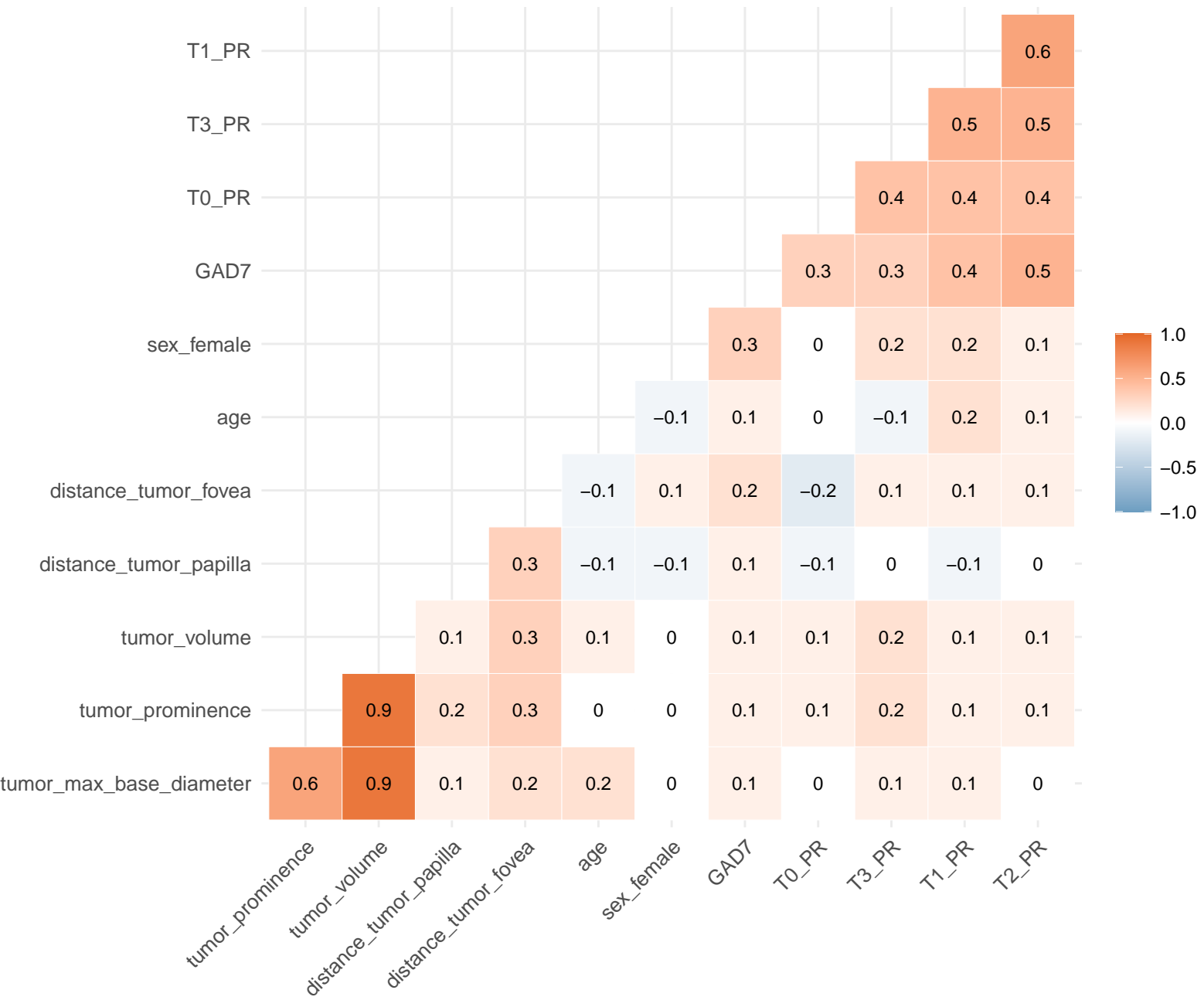

Supplement: Supplementary file 12 — Additional file 12. Heatmap showing spearman’s rank correlation coefficients between all variables regrading a given subscale for timepoints T0-T3. [file 13014_2021_1902_MOESM12_ESM.pdf]

Heatmap Role functioning T0–T3 (RF)

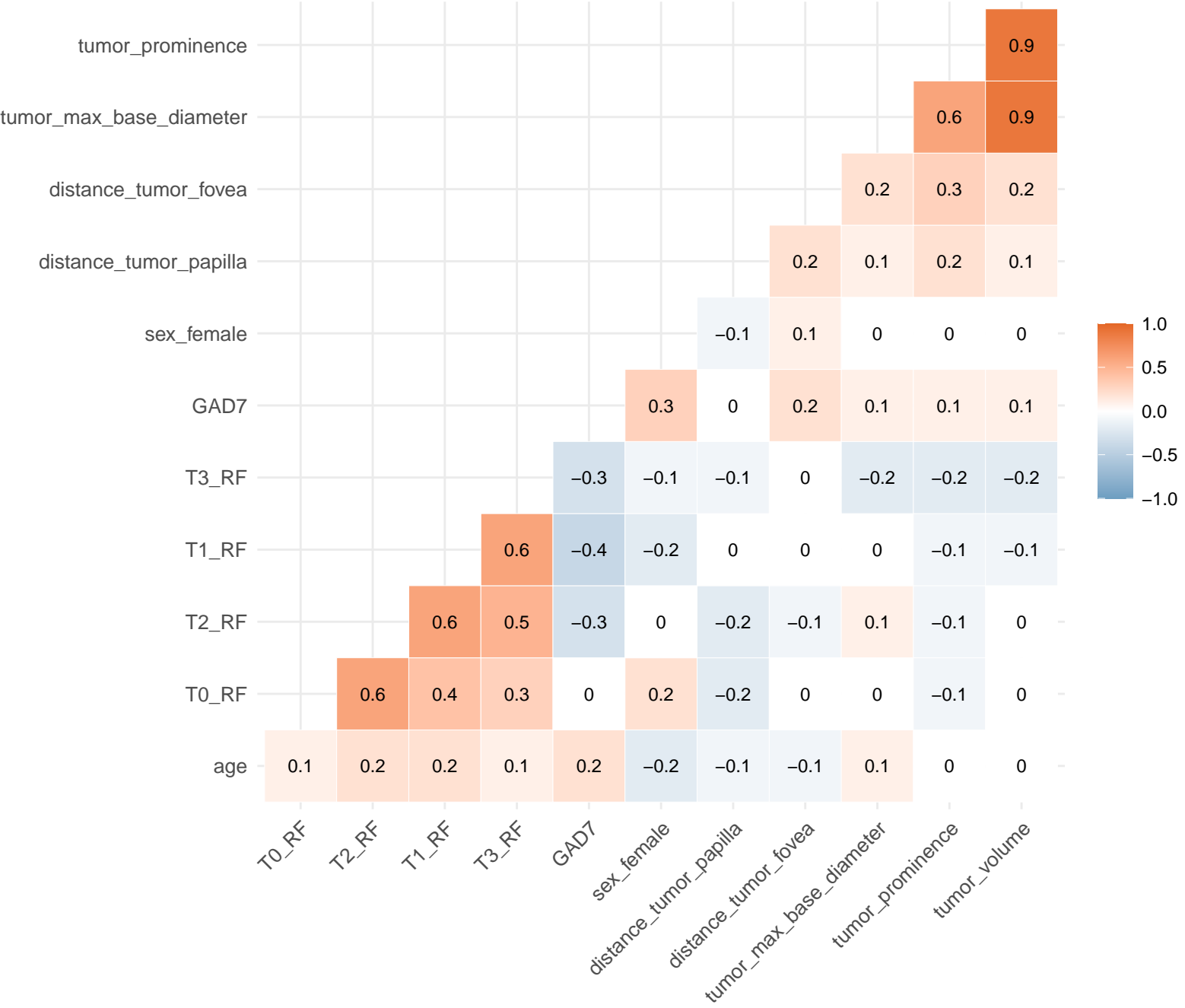

Supplement: Supplementary file 13 — Additional file 13. Heatmap showing spearman’s rank correlation coefficients between all variables regrading a given subscale for timepoints T0-T3. [file 13014_2021_1902_MOESM13_ESM.pdf]

Heatmap Social functioning T0–T3 (SF)

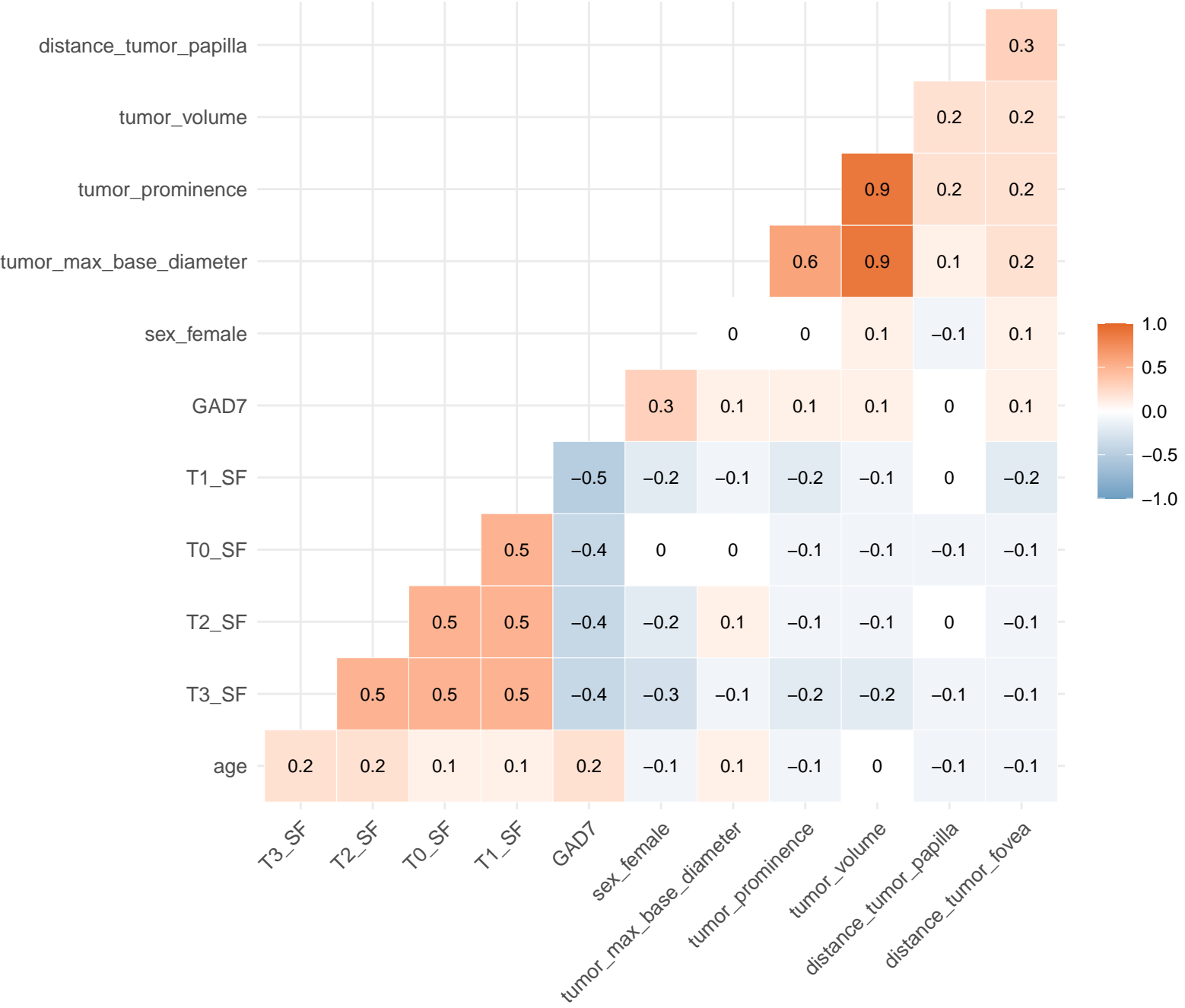

Supplement: Supplementary file 14 — Additional file 14. Heatmap showing spearman’s rank correlation coefficients between all variables regrading a given subscale for timepoints T0-T3. [file 13014_2021_1902_MOESM14_ESM.pdf]

Heatmap Visual impairment T0–T3 (VI)

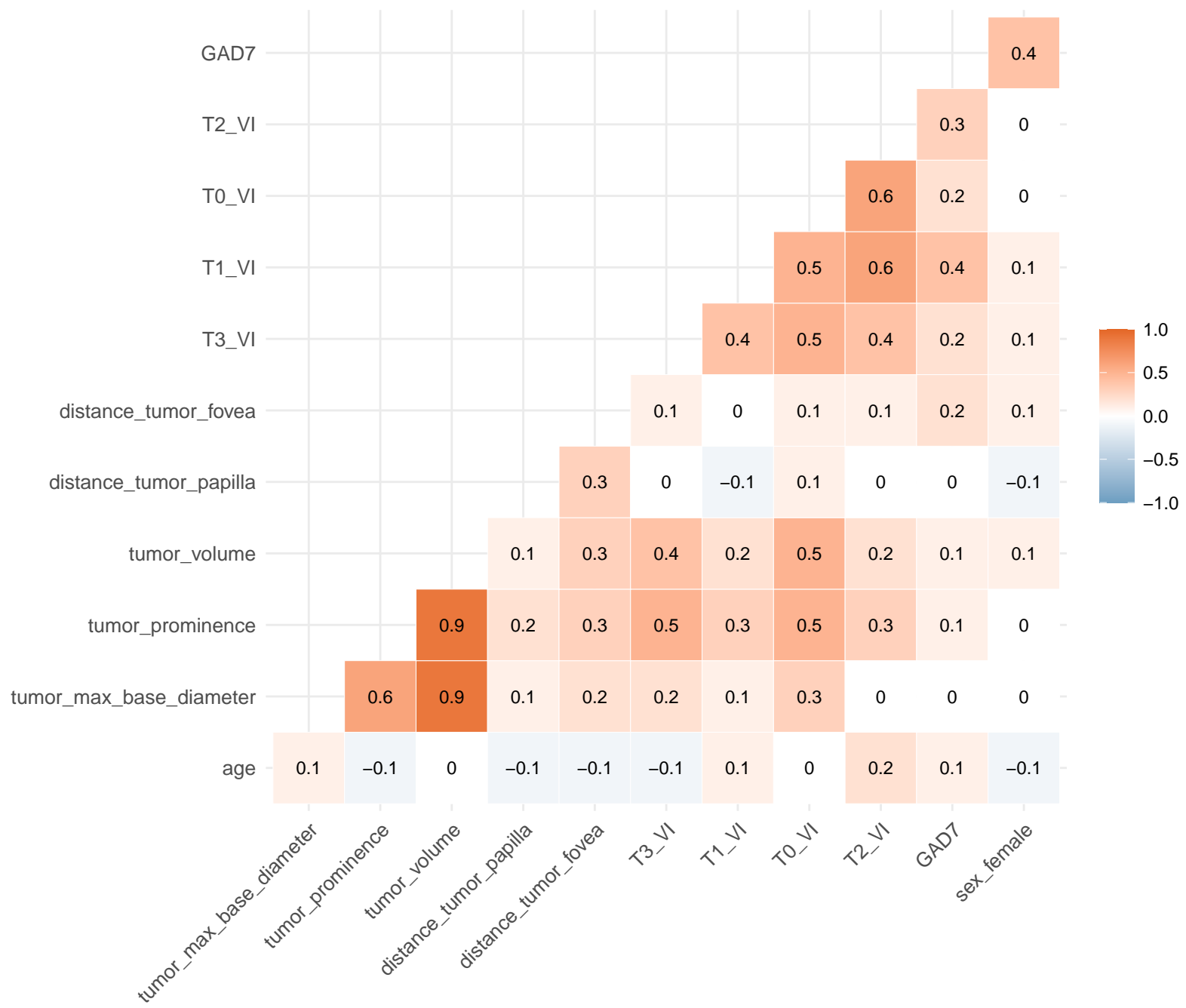

Supplement: Supplementary file 15 — Additional file 15. Heatmap showing spearman’s rank correlation coefficients between all variables regrading a given subscale for timepoints T0-T3. [file 13014_2021_1902_MOESM15_ESM.pdf]

Heatmap Worry about recurrence T0–T3 (WAR)

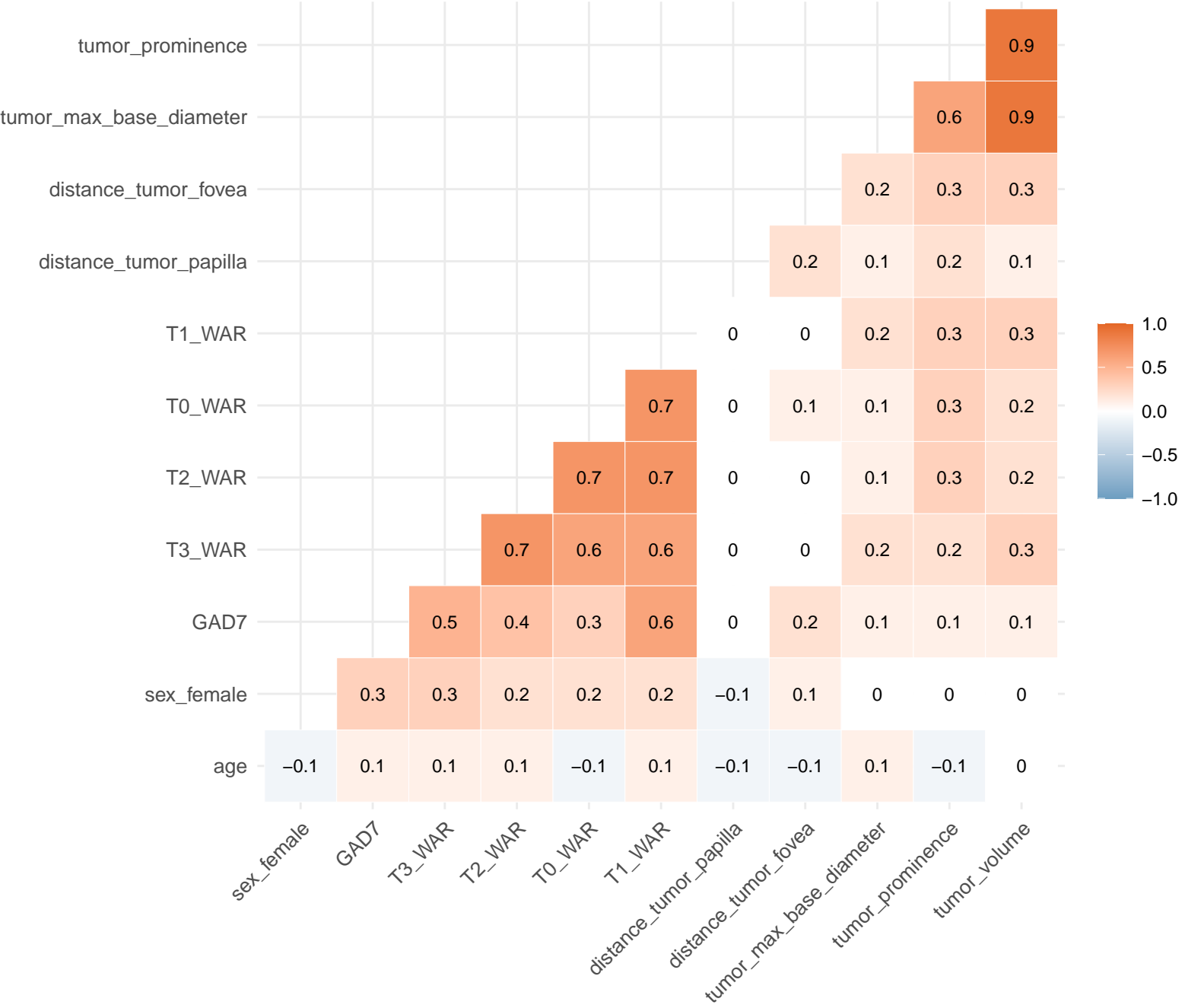

Supplement: Supplementary file 16 — Additional file 16. Final multiple linear regression models for all endpoints of QLQ-C30 and QLQ-OPT30. [file 13014_2021_1902_MOESM16_ESM.pdf]
