# Supplementary material for: Quality of life and treatment-related burden during ocular proton therapy: a prospective trial of 131 patients with uveal melanoma
Source: Radiat Oncol. 2021 Sep 8;16:174. doi: 10.1186/s13014-021-01902-6 (PMC8425039; doi:10.1186/s13014-021-01902-6)

Global Health (GH) by GAD7 and timepoint

Marginal estimates of males and females from multiple linear mixed regression:

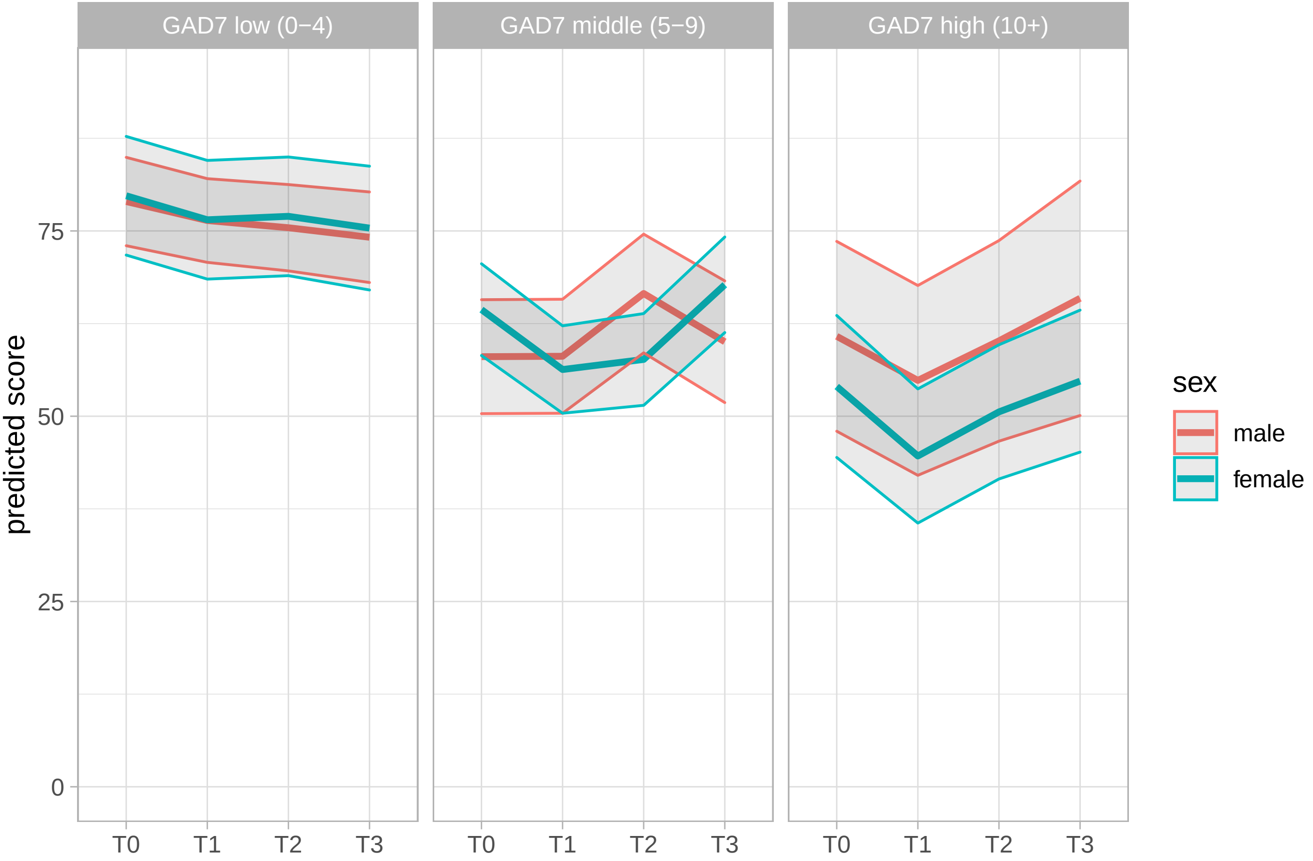

Supplement: Supplementary file 18 — Additional file 18. Contrast analyses of estimated marginal means between male and female patients by GAD-7 category for selected endpoints of QLQ-C30 and QLQ-OPT30. [file 13014_2021_1902_MOESM18_ESM.pdf]
