# Supplementary material for: Quality of life and treatment-related burden during ocular proton therapy: a prospective trial of 131 patients with uveal melanoma
Source: Radiat Oncol. 2021 Sep 8;16:174. doi: 10.1186/s13014-021-01902-6 (PMC8425039; doi:10.1186/s13014-021-01902-6)
Supplement: Supplementary file 20 — Additional file 20. Visualization of marginal estimates of outcome values with 95% confidence interval at different time points (T0-T3) by sex and GAD-7 subcategory. [file 13014_2021_1902_MOESM20_ESM.pdf]

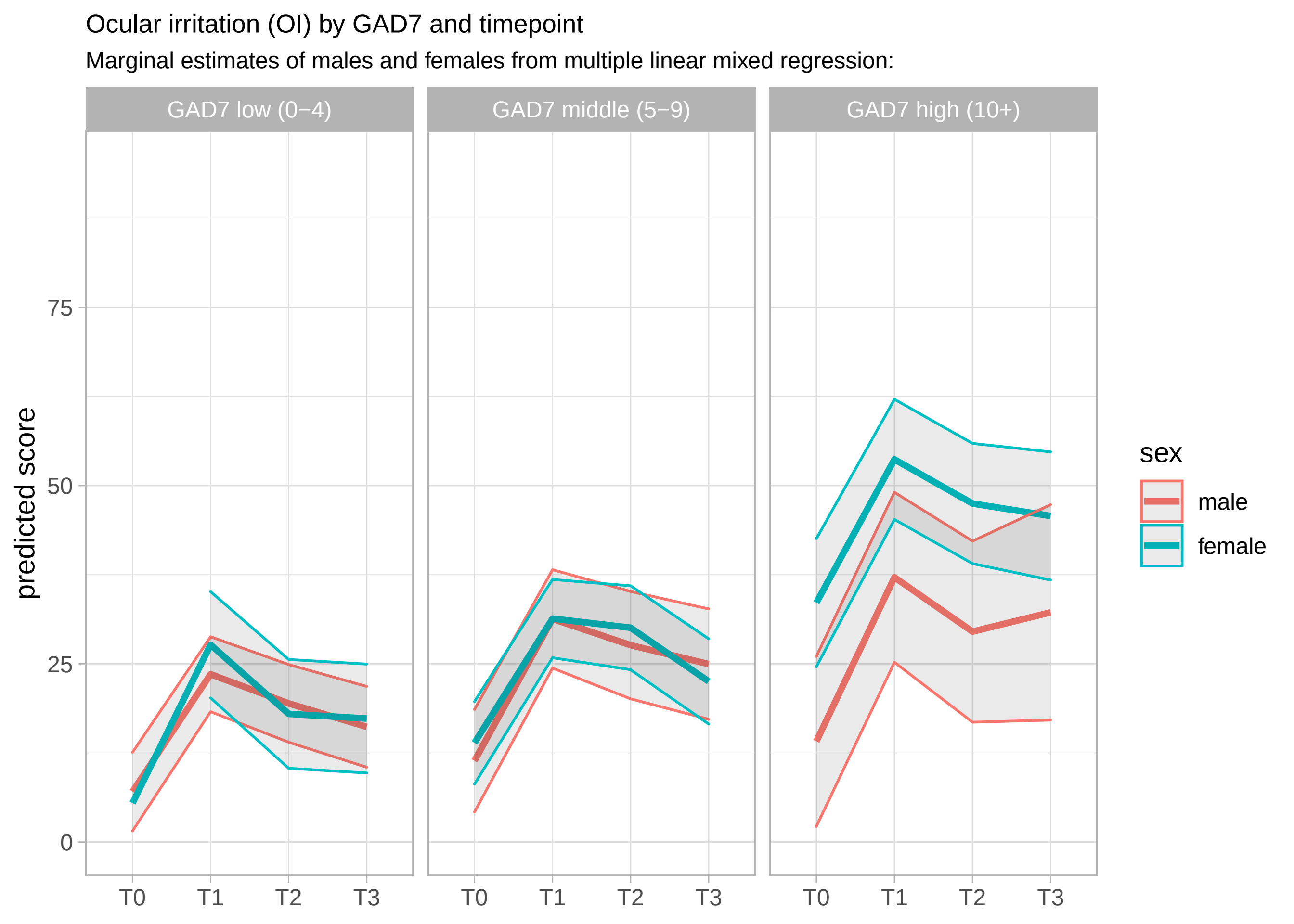

Ocular irritation (OI) by GAD7 and timepoint
Marginal estimates of males and females from multiple linear mixed regression:
GAD7 low (0−4)
GAD7 middle (5−9)
GAD7 high (10+)
predicted score
0
25
50
75
T0
T1
T2
T3
sex
male
female
