# Supplementary material for: Quality of life and treatment-related burden during ocular proton therapy: a prospective trial of 131 patients with uveal melanoma
Source: Radiat Oncol. 2021 Sep 8;16:174. doi: 10.1186/s13014-021-01902-6 (PMC8425039; doi:10.1186/s13014-021-01902-6)
Supplement: Supplementary file 21 — Additional file 21. Visualization of marginal estimates of outcome values with 95% confidence interval at different time points (T0-T3) by sex and GAD-7 subcategory. [file 13014_2021_1902_MOESM21_ESM.docx]

**Final multiple linear mixed regression model for**

**Emotional functioning (EF) T0-T3**

(random intercept model, n=129 individuals, 466 measures)

|  |  | | | |
| --- | --- | --- | --- | --- |
|  | *β* | *95%CI* | *p* | *Partial R^2^* |
| (Intercept) | 73.54 | 61.66 – 85.41 | <0.001 |  |
| age | 0.24 | 0.05 – 0.43 | 0.014 | 0.03 |
| sex: male |  |  |  |  |
| T0*GAD7 | -3.51 | -4.43 – -2.60 | <0.001 | 0.13 |
| T1*GAD7 | -3.67 | -4.59 – -2.75 | <0.001 | 0.14 |
| T2*GAD7 | -2.39 | -3.34 – -1.44 | <0.001 | 0.06 |
| T3*GAD7 | -2.36 | -3.45 – -1.26 | <0.001 | 0.04 |
| sex: female |  |  |  |  |
| T0*GAD7 | -4.09 | 4.84 – -3.34 | <0.001 | 0.23 |
| T1*GAD7 | -3.96 | -4.68 – -3.23 | <0.001 | 0.24 |
| T2*GAD7 | -3.62 | -4.36 – -2.89 | <0.001 | 0.20 |
| T3*GAD7 | -3.07 | -3.83 – -2.32 | <0.001 | 0.14 |
| total | 0.42 | | | |

**Final multiple linear mixed regression model for**

**Problems with exterior aspect (PA) T0-T3**

(random intercept model, n=129 individuals, 458 measures)

|  |  | | | |
| --- | --- | --- | --- | --- |
|  | *β* | *95%CI* | *p* | *Partial R^2^* |
| (Intercept) | 17 | 61.66 – 85.41 | 0.007 |  |
| age | -0.12 | 0.05 – 0.43 | 0.2 | 0.005 |
| sex: male |  |  |  |  |
| T0*GAD7 | -0.28 | -1.4 - 0.81 | <0.001 | 0.001 |
| T1*GAD7 | 1.7 | 0.62 - 2.8 | <0.001 | 0.023 |
| T2*GAD7 | 1.0 | -0.24 - 2.2 | <0.001 | 0.006 |
| T3*GAD7 | 0.82 | -0.58 - 2.2 | <0.001 | 0.003 |
| sex: female |  |  |  |  |
| T0*GAD7 | -0.23 | -1.1 - 0.66 | <0.001 | 0.001 |
| T1*GAD7 | 2.5 | 1.6 - 3.3 | <0.001 | 0.075 |
| T2*GAD7 | 3.0 | 2.1 - 3.9 | <0.001 | 0.098 |
| T3*GAD7 | 2.3 | 1.4 - 3.2 | <0.001 | 0.061 |
| total | 0.191 | | | |

**Final multiple linear mixed regression model for**

**Ocular irritation (OI) T0-T3**

(random intercept model, n=129 individuals, 465 measures)

|  |  | | | |
| --- | --- | --- | --- | --- |
|  | *β* | *95%CI* | *p* | *Partial R^2^* |
| (Intercept) | 14 | 4.4 – 23 | 0.004 |  |
| age | -0.06 | -0.20 – 0.08 | 0.4 | 0.002 |
| tumor_prominence | 0.48 | -0.16 – 1.1 | 0.14 | 0.008 |
| sex: male |  |  |  |  |
| T0*GAD7 | -0.17 | -0.92 - 0.57 | <0.001 | 0.001 |
| T1*GAD7 | 2.6 | 1.8 – 3.3 | <0.001 | 0.103 |
| T2*GAD7 | 1.8 | 1.0 - 2.6 | <0.001 | 0.048 |
| T3*GAD7 | 1.8 | 0.82 - 2.7 | <0.001 | 0.029 |
| sex: female |  |  |  |  |
| T0*GAD7 | 0.85 | 0.23 – 1.5 | <0.001 | 0.018 |
| T1*GAD7 | 2.9 | 2.3 - 3.5 | <0.001 | 0.195 |
| T2*GAD7 | 2.6 | 2.0 - 3.2 | <0.001 | 0.159 |
| T3*GAD7 | 2.1 | 1.5 - 2.7 | <0.001 | 0.098 |
| total | 0.329 | | | |

**Final multiple linear mixed regression model for**

**Global health (GH) T0-T3**

(random intercept model, n=129 individuals, 463 measures)

|  |  | | | |
| --- | --- | --- | --- | --- |
|  | *β* | *95%CI* | *p* | *Partial R^2^* |
| (Intercept) | 81 | 71 – 91 | <0.001 |  |
| age | -0.01 | -0.17 – 0.14 | 0.9 | 0.000 |
| tumor_prominence | 0.48 | -0.16 – 1.1 | 0.14 | 0.008 |
| sex: male |  |  |  |  |
| T0*GAD7 | -2.4 | -2.7 – -1.4 | <0.001 | 0.085 |
| T1*GAD7 | -2.7 | -3.5 – -1.9 | <0.001 | 0.101 |
| T2*GAD7 | -1.8 | -2.6 – -0.93 | <0.001 | 0.084 |
| T3*GAD7 | -2.2 | -3.1 – -1.2 | <0.001 | 0.040 |
| sex: female |  |  |  |  |
| T0*GAD7 | -2.1 | -3.2 – -1.6 | <0.001 | 0.091 |
| T1*GAD7 | -2.9 | -3.6 – -2.3 | <0.001 | 0.187 |
| T2*GAD7 | -2.6 | -3.2 - -2.0 | <0.001 | 0.147 |
| T3*GAD7 | -1.9 | -2.6 - -1.3 | <0.001 | 0.079 |
| total | 0.290 | | | |
